# Fungal oxidative stress tolerance depends on peroxiredoxin PrxA-mediated redox signaling to mitochondrial cytochrome *c* peroxidase Ccp1

**DOI:** 10.1016/j.jbc.2026.113084

**Published:** 2026-04-27

**Authors:** Xiaofei Huang, Yan Gao, Bingzi Yu, Zehan Jia, Yiqing Luo, Mingxin Fu, Yuting Duan, Qianyun Bu, Xiaoying Li, Jing Wang, Xinyu Tan, Lingyan Guo, Jingyi Li, Yao Zhou, Xiaohui Zhang, Naoki Takaya, Shengmin Zhou

**Affiliations:** 1 State Key Laboratory of Bioreactor Engineering, School of Biotechnology, East China University of Science and Technology, Shanghai, China; 2 State Key Laboratory of Natural and Biomimetic Drugs, Peking University, Beijing, PR China; 3 Faculty of Life and Environmental Sciences, Microbiology Research Center for Sustainability, Tsukuba Institute for Advanced Research, University of Tsukuba, Tsukuba, Ibaraki, Japan

**Keywords:** oxidative stress tolerance, redox signaling, cytochrome *c* peroxidase, mitochondrial protection, peroxiredoxin

## Abstract

Oxidative stress represents a central challenge to cellular survival. Although multiple antioxidant enzymes participate in oxidative defense, peroxiredoxins (Prxs) have long been regarded as key determinants of oxidative stress tolerance. However, this view is largely based on the oxidative sensitivity of Prx-deficient mutants and lacks direct experimental evidence demonstrating that Prxs function as terminal antioxidant effectors determining cellular tolerance to oxidative stress. In this study, through transcriptomic screening combined with systematic genetic and functional analyses, we define the key effector within the oxidative defense system and identify the mitochondrial cytochrome *c* peroxidase Ccp1 as a core determinant of oxidative stress tolerance. In *Aspergillus nidulans*, the peroxiredoxin PrxA activates the oxidative-stress transcription factor NapA, mediating Ccp1 induction. Our results indicate that the apparent requirement for Prx in oxidative stress tolerance does not arise from its role as a terminal antioxidant effector, but instead reflects its function as an upstream redox signaling factor regulating activation of the key effector enzyme Ccp1. Further functional analyses show that loss of Ccp1 or catalytic inactivation leads to dissipation of mitochondrial membrane potential, compromised mitochondrial DNA integrity, and reduced iron–sulfur enzyme activity, thereby impairing cellular tolerance to oxidative stress. Redirecting other peroxidases to mitochondria functionally substitutes for Ccp1 and restores oxidative stress tolerance. Together, these findings demonstrate that mitochondria-targeted antioxidant protection mediated by Ccp1 acts as a key defensive process for oxidative stress tolerance, while mechanistically clarifying the functional role of PrxA as an upstream redox signaling factor within the oxidative defense network of this fungus.

Major eukaryotic defense mechanisms against H_2_O_2_ have been delineated (1). Catalases rapidly decompose peroxide (2); peroxiredoxins (Prxs) not only eliminate peroxides but also transduce redox signals *via* conformational and oxidative state changes, and exert chaperone functions through polymerization under specific conditions (3, 4, 5); the glutathione and thioredoxin cycles provide reducing power for antioxidant systems (6), and AP-1-like transcription factors of the NapA/Atf family induce antioxidant gene expression under oxidative stress (7). Together, these studies outline a multilayered framework involving signal perception, transcriptional activation, and effector functions. Yet several key questions regarding the species-specific characteristics of antioxidant mechanisms across different biological systems remain unresolved (8, 9, 10, 11).

*Aspergillus nidulans*, as a model filamentous fungus, exhibits a high oxidative metabolic rate and strong secretion capacity, with distinct subcellular redox heterogeneity (12, 13, 14, 15), making it an important model for dissecting the spatial regulation of redox homeostasis in eukaryotic cells. In this system, NapA has been established as the core transcription factor governing H_2_O_2_-induced antioxidant gene expression (16). Building on this regulatory scheme, our previous studies have progressively elucidated the molecular basis of oxidative stress defense in *A*. *nidulans*: peroxiredoxin PrxA was identified as an essential antioxidant factor required for H_2_O_2_ tolerance, whose function relies on the reducing equivalents provided by the thioredoxin/thioredoxin reductase (TrxA/TrxR) system (17); further work showed that NADPH, as the primary electron source for PrxA, maintains a dynamic intracellular balance that modulates cellular oxidative resistance, supplying the necessary reducing power for antioxidant systems while excess NADPH can paradoxically compromise defense function (18); on this basis, we revealed clear effector partitioning of antioxidant defense across developmental stages, in which PrxA dominates protection in dormant conidia and early growth phases, while catalase CatB acts by secretion to the cell surface during mature mycelial stages (15); and recent work further demonstrated that PrxA functions as a redox sensor, transmitting H_2_O_2_ signals through direct physical interaction with NapA and disulfide isomerization, whereas TrxA directly reduces and inactivates NapA in both the cytoplasm and nucleus, thereby establishing the PrxA–NapA–TrxA redox signaling axis that controls NapA activation and inactivation (19). Collectively, these studies indicate that oxidative stress defense in *A. nidulans* depends on a PrxA-centered redox signaling system that integrates reducing power supply, developmental stage-specific effector partitioning, and transcriptional control to achieve layered regulation of the H_2_O_2_ response. Although this regulatory framework has been established, how this signaling axis is translated into effective downstream antioxidant output remains to be clarified.

Here, using *A*. *nidulans*, we dissect how cells establish oxidative resistance under H_2_O_2_ stress. We show that the PrxA–NapA pathway forms the central regulatory axis and identify the mitochondrial cytochrome *c* peroxidase Ccp1 as its key effector. Our analyses demonstrate that mitochondria act not merely as vulnerable targets but as the primary sites of defense: both mitochondrial localization and catalytic activity of Ccp1 are required to sustain redox balance and metabolic integrity. These results thus provide a molecular basis for understanding subcellular localization-dependent antioxidant defense mechanisms in filamentous fungi.

## Results

### Low-dose H_2_O_2_ priming enhances survival under lethal challenge

**Figure 1:**
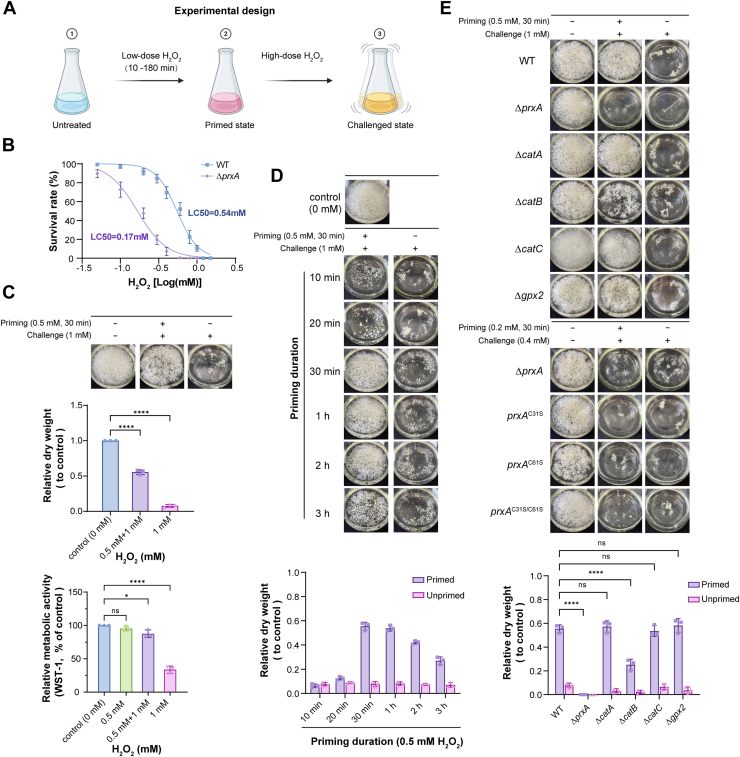
**Low-doseH_2_O_2_priming enhances oxidative stress tolerance in*****A. nidulans*****, and PrxA is required for adaptive resistance.***A*, experimental design of the priming–challenge assay. Cultures were exposed to 0.5 mM H_2_O_2_ for 10 to 180 min to establish a primed state and subsequently challenged with 1 mM H_2_O_2_. *B*, Dose–response curves and LC50 determination of WT and Δ*prxA* strains under increasing concentrations of H_2_O_2_. Conidia were cultured in liquid minimal medium for 4.5 h before the addition of H_2_O_2_ and further incubation for 12 h. Survival rates were calculated based on mycelial dry weight. LC50 values were determined by nonlinear regression analysis. Data represent mean ± SD from three independent biological replicates. *C*, effects of priming on oxidative stress tolerance. Hyphae were untreated (control), primed (0.5 mM H_2_O_2_ for 30 min followed by 1 mM H_2_O_2_), or directly exposed to 1 mM H_2_O_2_. Hyphal morphology after 12 h of challenge, dry-weight measurements from the same cultures, and WST-1 reduction 1 h after challenge are shown. Data represent the mean ± SD from three independent biological replicates (n = 3). Statistical significance was assessed using one-way ANOVA with Dunnett’s multiple-comparison test (∗∗∗∗*p* < 0.0001; ns, not significant). *D*, time-dependent effects of priming. Cultures were primed with 0.5 mM H_2_O_2_ for 10, 20, or 30 min, 1 h, 2 h, or 3 h and then challenged with 1 mM H_2_O_2_. Representative hyphal morphology after challenge and corresponding dry-weight measurements are shown. Data represent mean ± SD (n = 3). *E*, oxidative stress adaptation in antioxidant-enzyme deletion mutants. Wild-type and Δ*prxA*, Δ*catA*, Δ*catB*, Δ*catC*, and Δ*gpx2* strains were subjected to the 0.5 mM priming–1 mM challenge regimen. Hyphal morphology after challenge and dry-weight measurements from the same cultures are shown. For Δ*prxA, prxA*^C31S^, *prxA*^C61S^ and *prxA*^C31S/C61S^-expressing strains, an additional regimen (0.2 mM H_2_O_2_ for 30 min→0.4 mM H_2_O_2_) was also tested. Data represent mean ± SD (n = 3). Statistical significance was determined using two-way ANOVA with Dunnett’s multiple-comparison test (∗∗∗∗*p* < 0.0001; ns, not significant).

Microbial oxidative tolerance is often acquired through an adaptive phase triggered by mild, sublethal stimuli, which enhances survival under subsequent severe insults (20, 21). Such low-intensity oxidative cues are common in natural and host environments, where transient, nonlethal ROS are generated during metabolic activity or early immune responses (1, 22). To examine whether *A. nidulans* exhibits this adaptive behavior, we established a two-step H_2_O_2_ treatment system (Fig. 1*A*): cultures were first exposed to 0.5 mM H_2_O_2_ for 10 to 180 min to induce a primed state, followed by a lethal challenge of 1 mM H_2_O_2_. This design mimics gradual oxidative escalation and allows systematic evaluation of tolerance. The selection of priming and challenge concentrations was guided by dose–response analysis (Fig. 1*B*). Specifically, 0.5 mM H_2_O_2_ was chosen as a near-threshold sublethal priming dose, allowing cells to perceive oxidative stress and mount an adaptive response without severe growth inhibition. In contrast, 1 mM H_2_O_2_ was selected as a strong challenge dose that remains below the fully lethal range. Under this design, if priming fails to induce protection, the sequential priming-plus-challenge treatment imposes cumulative oxidative stress and results in a more pronounced inhibitory effect; if priming is effective, a clear adaptive benefit can be detected. By contrast, use of an excessively lethal challenge dose would collapse this distinction, as both primed and non-primed cells would be similarly overwhelmed. Pretreatment with 0.5 mM H_2_O_2_ for 30 to 60 min markedly improved survival under 1 mM challenge, as primed hyphae maintained robust growth (Fig. 1*C*, top) and showed significantly higher dry weight than untreated controls (Fig. 1*C*, middle). Metabolic assessment using WST-1 reduction showed that 1 mM H_2_O_2_ strongly inhibited mitochondrial activity, whereas 30 to 60 min priming preserved reduction capacity at levels comparable to untreated samples and substantially higher than non-primed controls (Fig. 1*C*, bottom). Time-course analysis revealed a clear temporal dependency: minimal protection at 10 min, maximal adaptation at 30 to 60 min, and declining effects after 2 to 3 h (Fig. 1*D*, top and bottom), indicating an optimal priming window. Primed cells also cleared exogenous H_2_O_2_ more rapidly (Fig. S1*A*, colorimetric assay), and accumulated less intracellular H_2_O_2_ during subsequent exposure (Fig. S1*B*, BES-H_2_O_2_-Ac fluorescent probe). Together, these findings demonstrate that mild H_2_O_2_ pretreatment effectively induces oxidative tolerance in *A. nidulans*, enhancing growth and metabolic resilience under otherwise lethal oxidative stress.

### PrxA is essential for oxidative stress adaptation in *A. nidulans*

The enhanced H_2_O_2_ clearance observed after priming suggested that oxidative tolerance may depend on specific peroxide-scavenging systems. To identify the key effectors, we examined mutants defective in major H_2_O_2_-removal pathways—including the peroxiredoxin *prxA*, the catalases (*catA*, *catB*, *catC*), and the glutathione peroxidase *gpx2*—under a defined priming–challenge regimen (0.5 mM H_2_O_2_ priming followed by 1 mM H_2_O_2_ challenge), representing the principal tiers of eukaryotic antioxidant defense (23). Within the catalase family, Δ*catB* showed partial recovery upon priming, but both hyphal mass and dry weight were markedly lower than those of the wild type (Fig. 1*E*, upper panels), indicating that CatB plays only a supportive role. By contrast, Δ*catA*, Δ*catC*, and Δ*gpx2* exhibited no priming-dependent differences compared with the wild type (Fig. 1*E*, upper panels). Under the standard 0.5 mM priming → 1 mM challenge regimen, Δ*prxA* exhibited profound hypersensitivity to 1 mM H_2_O_2_ (Fig. 1*E*, upper panels), making it impossible to determine whether priming could confer any adaptive benefit. Therefore, a milder oxidative regimen (0.2 mM priming → 0.4 mM challenge) was applied for Δ*prxA*, as guided by its increased sensitivity revealed by dose–response analysis (Fig. 1*B*). Even under these alleviated conditions, Δ*prxA* showed no priming-dependent improvement in morphology or biomass (Fig. 1*E*, lower panels), demonstrating that PrxA is indispensable for oxidative adaptation. Together, these results indicate that adaptive oxidative tolerance in *A. nidulans* critically depends on PrxA, with CatB contributing modestly, whereas CatA, CatC, and Gpx2 have a negligible impact.

### PrxA activity rather than expression level governs adaptive tolerance

**Figure 2:**
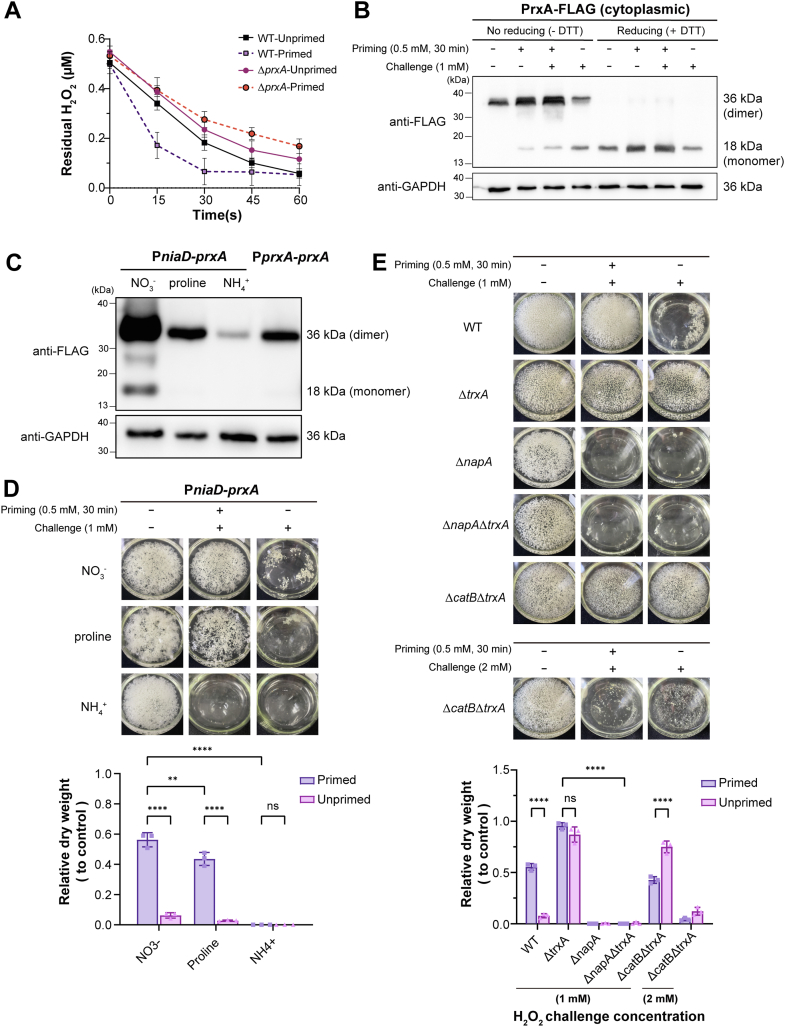
**PrxA and its associated antioxidant systems play key roles in oxidative stress adaptation in*****A. nidulans***. *A*, H_2_O_2_ degradation assay. Wild-type and Δ*prxA* strains were grown for 4.5 h to the short-hyphal stage at 37 °C with shaking at 220 rpm. Cultures were then exposed to 1 mM H_2_O_2_ for 30 min with or without prior priming (0.5 mM H_2_O_2_, 30 min). Cell lysates were prepared after treatment, and H_2_O_2_ (0.5 μM) degradation was measured in 100 μl reactions containing protein-normalized cell extracts and Amplex Red/HRP working solution. Data represent mean ± SD from three biological replicates (n = 3). *B*, redox-dependent molecular states of PrxA. The *prxA*-FLAG strain was grown for 4.5 h to the short-hyphal stage and subjected to priming (0.5 mM H_2_O_2_, 30 min) followed by challenge (1 mM H_2_O_2_). Protein extracts were analyzed under non-reducing (−DTT) or strongly reducing (+DTT) SDS buffer conditions using anti-FLAG immunoblotting, allowing detection of dimeric PrxA (36 kDa) and monomeric PrxA (18 kDa). GAPDH served as a loading control. Immediately after cell disruption, 25 mM N-ethylmaleimide (NEM) was added to trap free thiols and prevent post-lysis oxidation, ensuring the preservation of the *in vivo* redox state of PrxA. *C*, verification of PrxA expression levels in the P*niaD*-*prxA* strain under different nitrogen sources. P*niaD*-*prxA* strains were cultured for 4.5 h at 37 °C with shaking at 220 rpm in media containing nitrate, proline, or ammonium as the sole nitrogen source. PrxA abundance and dimer/monomer ratios were examined by anti-FLAG immunoblotting. The P*prxA*-*prxA* strain grown in nitrate MM served as a stable-expression control. GAPDH was used as a loading control. Immunoblots are representative of three independent biological experiments. *D*, effects of PrxA expression level on oxidative adaptation. P*niaD*-*prxA* strains were grown for 4.5 h under nitrate, proline, or ammonium as the sole nitrogen source at 37 °C with shaking at 220 rpm. Cultures were subjected to priming (0.5 mM H_2_O_2_, 30 min) and subsequently challenged with 1 mM H_2_O_2_ for 12 h. Hyphal pellet morphology and dry-weight measurements were quantitatively compared to assess adaptive tolerance. Images are representative of three independent biological replicates. Data indicate mean ± SD, n = 3. Statistical significance was determined using two-way ANOVA with Tukey’s test (∗∗∗∗*p* < 0.0001; ns, not significant). *E*, roles of TrxA and NapA pathways in PrxA-mediated oxidative adaptation. Wild-type, Δ*trxA*, Δ*napA*, Δ*napAΔtrxA*, and Δ*catBΔtrxA* strains were grown for 4.5 h to the short-hyphal stage at 37 °C with shaking at 220 rpm. Cultures were subjected to the same priming–challenge regimen (0.5 mM priming → 1 mM challenge → 12 h growth). For Δ*catBΔtrxA*, a higher challenge dose (2 mM H_2_O_2_) was applied to improve phenotypic resolution. All experiments were performed at 37 °C with shaking at 220 rpm. Hyphal morphology after challenge and corresponding dry-weight measurements from the same cultures are shown. Data indicate mean ± SD, n = 3. Statistical significance was determined using two-way ANOVA with Tukey’s test (∗∗∗∗*p* < 0.0001; ns, not significant).

Because low-dose priming enhanced peroxide-removal capacity in a PrxA-dependent manner, we examined whether PrxA directly contributes to H_2_O_2_ degradation. Crude extracts from primed wild-type cultures showed markedly increased H_2_O_2_ decomposition rates, whereas Δ*prxA* extracts exhibited uniformly low activity that was unaffected by priming (Fig. 2*A*), indicating that PrxA is the major driver of priming-induced peroxide scavenging. To determine whether this enhanced activity arises from increased *prxA* expression or redox-state changes, we analyzed PrxA molecular species under different oxidative conditions. To prevent post-lysis oxidation artifacts, N-ethylmaleimide (NEM) treatment was used to alkylate free thiols. After 30 min of 0.5 mM priming, the active dimeric form of PrxA (36 kDa) accumulated, with no significant change observed after subsequent exposure to 1 mM H_2_O_2_. In contrast, direct exposure to 1 mM H_2_O_2_ resulted in a significant reduction of the dimeric form compared to pre-treatment, with a notable increase in the monomeric form (18 kDa), representing the inactive hyperoxidized species (Fig. 2*B*). This pattern is consistent with the oxidation sensitivity of canonical 2-Cys Prxs, whose peroxidatic cysteine is readily hyperoxidized to sulfinic acid under high H_2_O_2_, preventing intermolecular disulfide formation and yielding monomers under denaturing conditions (10). Under strongly reducing conditions (SDS sample buffer containing DTT), the total amount of PrxA showed a significant increase after 30 min of 0.5 mM priming compared to pre-treatment, suggesting that priming induces an elevation in PrxA levels. To test whether this priming-induced increase in PrxA abundance is the primary determinant of enhanced antioxidant capacity, we engineered a tunable *prxA* expression system under the nitrogen-regulated *niaD* promoter, which is induced by nitrate, moderately active with proline, and repressed by ammonium (24, 25). As expected, PrxA levels were high, intermediate, or low under these respective nitrogen sources (Fig. 2*C*). In the priming–challenge assay, the low-expression strain (ammonium) almost completely lost adaptation, whereas intermediate and high expression strains (proline and nitrate) showed wild-type-like tolerance (Fig. 2*D*). These data indicate that PrxA is required for adaptation but functions in a threshold-dependent manner: insufficient expression abolishes tolerance, whereas expression above the threshold provides no additional benefit. Notably, strong induction of the P*niaD–*driven *prxA* construct resulted in pronounced accumulation of monomeric PrxA even in the absence of oxidative stress (Fig. 2*C*), indicating that overexpression *per se* predisposes PrxA to hyperoxidative inactivation or structural destabilization.

Previous studies have shown that some 2-Cys Prxs possess not only peroxidase activity but also chaperone-like functions that are independent of their catalytic cysteines (5, 26, 27, 28). To specifically test the requirement for catalytic activity in adaptive tolerance, we examined catalytic cysteine mutants previously generated in our laboratory (19). In canonical 2-Cys Prxs, the peroxidatic cysteine (C_p_) and resolving cysteine (C_r_) form an intermolecular disulfide during peroxide reduction (29). Accordingly, we analyzed the C31S mutant (lacking C_r_), the C61S mutant (lacking C_p_), and the C31S/C61S double mutant (lacking both C_p_ and C_r_); importantly, these mutations abolish peroxidase activity but do not affect the chaperone-like functions reported for some Prxs. All three mutants failed to acquire priming-induced tolerance and exhibited survival defects comparable to Δ*prxA* (Fig. 1*E*, bottom), demonstrating that both C_p_ and C_r_—thus disulfide-dependent redox relay—are essential for PrxA-mediated signaling to NapA. These findings indicate that oxidative adaptation relies on the redox-active, dimeric form of PrxA rather than on expression level or chaperone-like activity, and that low-dose priming likely prevents hyperoxidative inactivation to maintain PrxA in its active state.

### PrxA signals through NapA rather than TrxA to mediate oxidative adaptation

Given that PrxA protection relies on its catalytic activity, we next examined its mode of action in *Aspergillus*. PrxA has been proposed to act either by (i) TrxA-dependent peroxide reduction (30) or (ii) transmitting oxidative signals to NapA through a redox relay, supported by *in vivo* evidence of a direct PrxA-NapA interaction (19, 31). To determine which mechanism dominates during adaptation, we first tested the role of TrxA. If PrxA depended mainly on TrxA-driven detoxification, Δ*trxA* would be expected to show reduced tolerance. Instead, Δ*trxA* displayed markedly higher resistance to 1 mM H_2_O_2_ than the wild type, independent of priming (Fig. 2*E*), indicating that PrxA acts primarily through oxidative signal relay.

To test whether this relay requires NapA, we analyzed the Δ*napA* mutant. Under the original 0.5 mM priming–1 mM challenge regimen, Δ*napA* completely lost priming-induced protection and closely phenocopied the Δ*prxA* strain (Fig. 2*E*), preventing clear assessment of adaptive defects. Based on the LC50 values determined in our dose-response analysis (Fig. S2*A*), we adjusted the priming and challenge doses to 0.2 mM and 0.4 mM, respectively, for Δ*napA*. Under this calibrated regimen, Δ*napA* clearly failed to mount priming-induced adaptive tolerance, demonstrating that NapA is essential for PrxA-mediated oxidative adaptation. To further assess whether the elevated tolerance of Δ*trxA* also depends on NapA, we constructed a Δ*trxAΔnapA* double mutant. This strain showed a complete loss of hyper-tolerance, phenocopying the Δ*napA* single mutant (Figs. 2*E* and S2*B*), indicating that Δ*trxA* hyper-resistance requires NapA. These results, combined with our previous evidence that TrxA directly reduces and inactivates NapA (19), suggest that TrxA deficiency impairs the reductive inactivation of NapA and promotes the accumulation of oxidized PrxA, which together act as a redox relay to sustain the activation of NapA-dependent antioxidant defenses and confer hyper-resistance to H_2_O_2_.

To identify NapA effector outputs, we examined the Δ*trxAΔcatB* mutant, given that CatB is a proposed NapA target (16, 19). At 1 mM H_2_O_2_, this strain maintained strong tolerance, only slightly weaker than Δ*trxA*. Even at 2 mM H_2_O_2_, its growth remained significantly higher than the wild type. Thus, although CatB is NapA-regulated, it is not the major effector of the PrxA–NapA axis. Collectively, these findings indicate that NapA mediates oxidative adaptation through additional downstream effectors beyond CatB, although the full set of NapA targets remains to be defined.

### Transcriptome analysis reveals a low-dose induction–high-dose attenuation oxidative adaptation network

**Figure 3:**
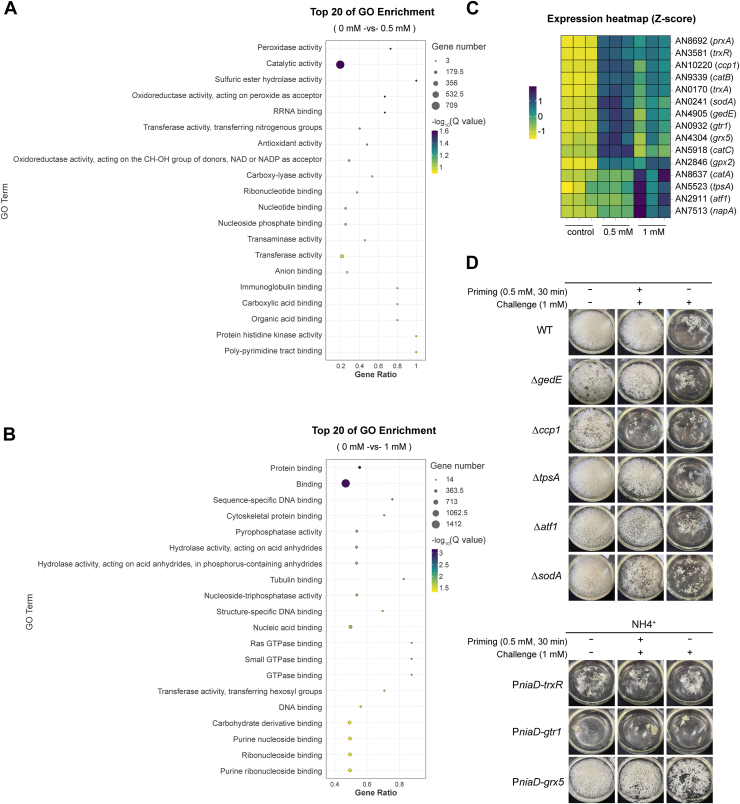
**Transcriptomic identification and functional analysis of candidate oxidative-adaptation genes.***A* and *B*, GO enrichment of differentially expressed genes (DEGs) under low- and high-dose H_2_O_2_. *A*, top 20 enriched GO terms for DEGs in the 0 mM vs 0.5 mM comparison. Bubble size indicates gene number; color reflects enrichment significance (–log_10_(q value)); The x-axis indicates the gene ratio (DEGs in a GO term/total DEGs). Low-dose H_2_O_2_ preferentially enriched antioxidant- and oxidoreductase-related functions. *B*, top 20 enriched GO terms for DEGs in the 0 mM vs 1 mM comparison. High-dose exposure strongly enriched protein-binding and sequence-specific DNA-binding functions, indicating intensified transcriptional reprogramming. *C*, expression profiles of antioxidant-related candidate genes. Wild-type *A. nidulans* was exposed to control, 0.5 mM, or 1 mM H_2_O_2_ and subjected to RNA-seq analysis. Genes with antioxidant activity enriched at low dose were combined with oxidative-stress-related GSEA terms to yield 15 candidates (including *prxA*, *trxR*, *ccp1*, *catB*, *trxA*, *sodA*, *gedE*, *gtr1*, *grx5*, *catC*, *gpx2*, *catA*, *tpsA*, *atf1*, and *napA*). The heatmap displays Z-score expression values (*yellow, high; purple, low*). *D*, functional assays of candidate genes under the priming–challenge regimen. (*Upper*) Deletion strains (Δ*gedE*, Δ*ccp1*, Δ*tpsA*, Δ*atf1*, Δ*sodA*) were pre-cultured for 4.5 h and subjected to priming (0.5 mM H_2_O_2_, 30 min) followed by challenge (1 mM H_2_O_2_) for 12 h. Growth phenotypes reflect the contribution of each gene to oxidative adaptation. (*Lower*) P*niaD*-driven overexpression strains (P*niaD-trxR*, P*niaD-gtr1*, P*niaD-grx5*) were pre-cultured for 4.5 h in NH_4_^+^ medium (repressive condition for P*niaD*) and subjected to the same priming–challenge treatments. Growth phenotypes indicate whether increased expression enhances oxidative tolerance. Quantification of mycelial dry weight corresponding to these phenotypes is shown in Fig. S2*C*.

To identify the key oxidative-response factors downstream of NapA, we next performed RNA-seq on wild-type mycelia under untreated, 0.5 mM, and 1 mM H_2_O_2_. Principal Component Analysis (PCA) (Fig. S3*A*) showed tight clustering within replicates and clear separation between treatments, indicating distinct transcriptional states. Correlation heatmaps (Fig. S3*B*) confirmed high reproducibility. Differential expression analysis (Table S1, sheet 1 and Fig. S3, *C*–*H*) revealed strong dose-dependent remodeling. Compared with the untreated sample, 0.5 mM H_2_O_2_ induced 733 upregulated and 1069 downregulated genes, whereas 1 mM induced far broader changes (1422 up; 3053 down). Thus, high-dose stress triggered markedly larger transcriptomic shifts. Volcano plots and clustering further showed that the 1 mM profile diverged substantially more from the control than did the 0.5 mM profile. GO enrichment (Table S1 sheet 2 and Fig. 3*A*) showed that low-dose H_2_O_2_ primarily enriched peroxidase, oxidoreductase, and antioxidant activities, reflecting activation of oxidative signaling and detoxification. High-dose treatment (Table S1 sheet 3 and Fig. 3*B*) enriched protein-binding and sequence-specific DNA-binding terms, consistent with intensive transcriptional reprogramming under severe stress.

Because low-dose priming enhanced H_2_O_2_ degradation capacity, suggesting selective activation of antioxidant defenses, we focused on genes responsive to mild oxidative cues. Representative genes from the low-dose–enriched “antioxidant activity” category included AN3581 (*trxR*), AN9339 (*catB*), AN10220 (*ccp1*), AN2846 (*gpx2*), AN5918 (*cta1*), AN4905 (*gedE*), AN8692 (*prxA*), AN0241 (*sodA*), AN0170 (*trxA*), and AN8637 (*catA*). To capture genes with modest but relevant effects, we additionally performed GSEA (Gene Set Enrichment Analysis) on oxidative stress–related pathways (“response to oxidative stress” and “response to oxygen-containing compound”, Table S1 sheet 4), identifying AN0932 (*gtr-1*), AN7513 (*napA*), AN4304 (*grx5*), AN5523 (*tpsA*), and AN2911 (*atf1*). qRT-PCR validation of all 15 genes (Fig. S4) strongly agreed with RNA-seq (Table S1 sheet 5 and Fig. 3*C*): most genes peaked at 0.5 mM and declined at 1 mM H_2_O_2_, whereas a subset remained highly expressed. Overall, these data define a characteristic “low-dose induction–high-dose attenuation” transcriptional signature underlying oxidative adaptation in *A. nidulans*.

### Ccp1 is the key NapA-Dependent effector driving adaptive oxidative resistance

To evaluate the roles of transcriptome-identified candidates in oxidative adaptation, we attempted to delete *ccp1*, *gedE*, *sodA*, *tpsA*, *atf1*, *trxR*, *gtr1*, and *grx5*. Stable knockout strains were successfully obtained for *ccp1*, *gedE*, *sodA*, *tpsA*, and *atf1* (Figs. 3*D*, top and S2*C*), whereas repeated attempts to delete *trxR*, *gtr1*, and *grx5* consistently failed, suggesting that these genes are essential for growth or basal metabolism and therefore cannot be assessed through conventional knockout approaches. To probe whether these essential genes contribute to adaptive resistance, we used a *niaD* promoter-replacement system to repress their expression under ammonium and examined tolerance in the 0.5 mM priming–1 mM challenge assay (Fig. 3*D*, bottom). Under repressive conditions, P*niaD-trxR* and P*niaD-gtr1* exhibited poor basal growth, leaving their involvement in adaptation unresolved. By contrast, P*niaD-grx5* displayed wild-type basal growth, H_2_O_2_ tolerance, and priming-induced benefits, indicating that *grx5* does not participate in oxidative adaptation. Among all deletion strains, Δ*ccp1* showed the most severe defect (Fig. 3*D*, top): it almost completely lost priming-induced protection, whereas Δ*gedE*, Δ*sodA*, Δ*tpsA*, and Δ*atf1* behaved similarly to the wild type. These data identify Ccp1 as the only transcriptome-derived candidate with a decisive role in adaptive oxidative resistance.

**Figure 4:**
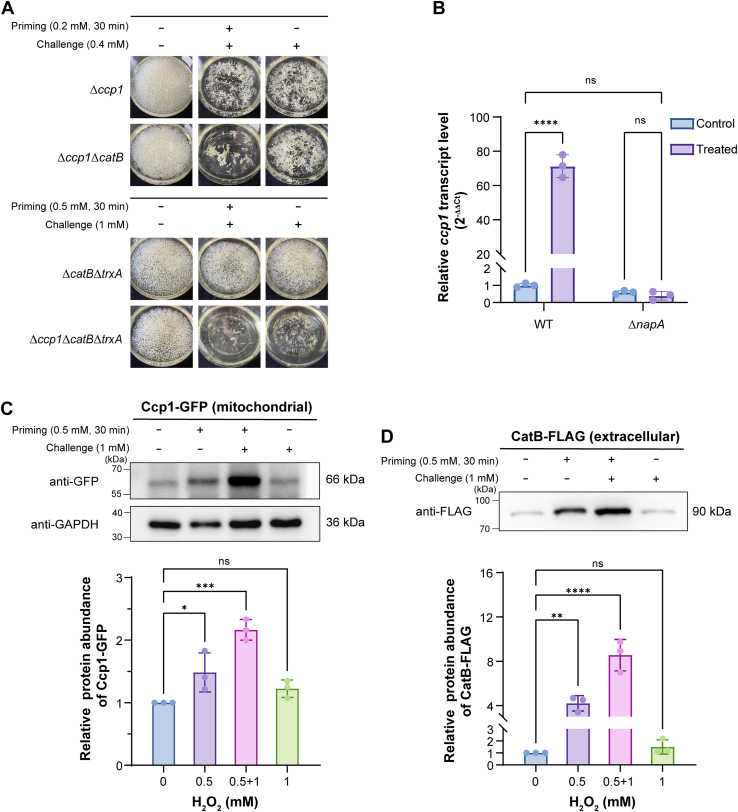
**Functional validation of Ccp1 as the principal NapA-dependent effector of oxidative adaptation.***A*, phenotypic analyses of Ccp1 and CatB in adaptive resistance. Hyphae of the indicated strains were grown in MM for 4.5 h to the short-hyphal stage and subjected to priming–challenge treatments. Upper panel: Comparison of Δ*ccp1* and Δ*ccp1*Δ*catB* under priming with 0.2 mM H_2_O_2_ for 30 min followed by challenge with 0.4 mM H_2_O_2_ for 12 h. Lower panel: Comparison of Δ*catB*Δ*trxA* and Δ*ccp1*Δ*catB*Δ*trxA* under priming with 0.5 mM H_2_O_2_ for 30 min followed by challenge with 1 mM H_2_O_2_ for 12 h. Oxidative tolerance was evaluated by hyphal-pellet number and size. Quantification of mycelial dry weight corresponding to these phenotypes is shown in Figure S2D. *B*, NapA-dependent induction of *ccp1* transcription. qRT-PCR quantification of *ccp1* transcript levels in WT and Δ*napA* after treatment with 0.5 mM H_2_O_2_ for 15 min. Data were analysed using the 2^−ΔΔCt^ method, normalized to *actin* expression within each sample, and expression levels compared to untreated conditions (control) in WT strain. Data indicate mean ± SD, n = 3. Statistical significance was determined using two-way ANOVA with Tukey’s test (∗∗∗∗*p* < 0.0001; ns, not significant). *C*, Ccp1-GFP and CatB-FLAG abundance under different oxidative regimes. The *ccp1-gfp* strain was cultured for 5 h and exposed to 0, 0.5 or 1 mM H_2_O_2_ for 30 min, or primed with 0.5 mM H_2_O_2_ for 30 min followed by 1 mM H_2_O_2_ for 30 min (“0.5 + 1”). Whole-cell extracts were analyzed by anti-GFP immunoblotting; anti-GAPDH served as a loading control. Quantification of band intensity is shown on the lower. Data represent mean ± SD (n = 3 independent biological replicates). *D*, abundance of extracellular CatB-FLAG under the same oxidative treatments. The *catB*-FLAG strain was treated as in (*C*). Culture supernatants were concentrated through a 10 kDa ultrafiltration membrane, and CatB-FLAG was detected by anti-FLAG immunoblotting. Quantification of band intensity is shown on the lower. Data represent mean ± SD (n = 3 independent biological replicates). Statistical significance was determined using one-way ANOVA with Dunnett’s multiple-comparison test (∗∗∗∗*p* < 0.0001; ns, not significant).

To clarify the relative contributions of Ccp1 and CatB during adaptation, we compared Δ*ccp1* and Δ*ccp1*Δ*catB* under the conditions shown in Figure 4*A* (0.2–0.4 mM H_2_O_2_). Dry weight measurements (Fig. S2*D*) revealed that the two strains exhibited comparable basal tolerance, whereas the double mutant showed slightly reduced recovery after priming, indicating that CatB makes only a modest contribution. Importantly, CatB does not provide meaningful protection in the absence of Ccp1, reaffirming that Ccp1 is the dominant effector. Previous results showed that Δ*trxA*Δ*catB* retained unexpectedly high H_2_O_2_ tolerance, suggesting the presence of a major protective factor beyond CatB. To determine whether this enhanced resistance depends on Ccp1, we constructed a Δ*ccp1*Δ*catB*Δ*trxA* triple mutant. This strain completely lost basal tolerance and priming-induced adaptation (Figs. 4*A* and S2*D*), phenocopying Δ*ccp1*. Taken together, these results demonstrate that Ccp1 is the principal determinant of adaptive oxidative resistance, whereas CatB provides only limited auxiliary support.

At the molecular level, qRT-PCR showed that *ccp1* was strongly induced after priming, whereas this induction was almost completely lost in the Δ*napA* mutant (Fig. 4*B*), indicating that *ccp1* activation depends on NapA; this is consistent with previous reports that other antioxidant genes such as *catB* are likewise regulated by NapA (19).Protein-expression analysis further supported this pattern: within the same experimental framework, Ccp1*-*GFP increased under mild oxidative stress, reached its maximum under the priming-plus-challenge condition, and declined under higher H_2_O_2_ levels (Fig. 4*C*, with the immunoblot shown above and the corresponding quantification below). CatB*-*FLAG displayed a similar response pattern under the same treatments (Fig. 4*D*), showing induction at low dose, further enhancement after priming, and attenuation under stronger stress.Together, these results demonstrate that NapA coordinates the expression of multiple antioxidant factors in response to varying H_2_O_2_ intensities. Functionally, however, Ccp1 serves as the principal determinant of adaptive oxidative tolerance, whereas CatB contributes only as a secondary auxiliary factor.

### Ccp1 safeguards mitochondrial integrity during oxidative adaptation

**Figure 5:**
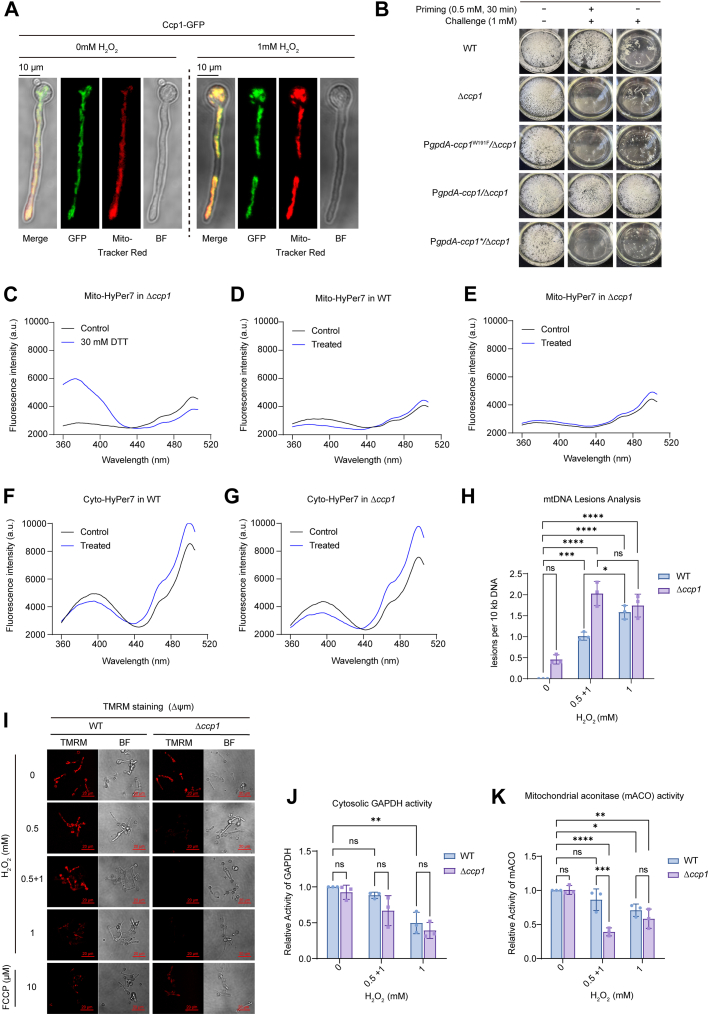
**Ccp1 protects mitochondria from oxidative damage.***A*, mitochondrial localization of Ccp1-GFP. Strains were cultured for 10 h until long hyphae formed and stained with 100 nM Mito-Tracker for 10 min under untreated or 1 mM H_2_O_2_ conditions. Fluorescence microscopy images are shown. Scale bar, 10 μm. Images are representative of three independent biological replicates. *B*, oxidative stress adaptation of Ccp1 variants. WT, Δ*ccp1*, P*gpdA*-*ccp1*, P*gpdA*-*ccp1*^W191F^, and P*gpdA*-*ccp1∗* strains were grown for 4.5 h, primed with 0.5 mM H_2_O_2_ for 30 min, and then challenged with 1 mM H_2_O_2_. Hyphal pellet morphology was examined after 12 h. Images are representative of three independent biological replicates. Quantification of mycelial dry weight corresponding to these phenotypes is shown in Figure S2E. *C–G*, HyPer7-based quantification of mitochondrial and cytosolic H_2_O_2_ levels in protoplasts. Protoplasts were generated from WT and Δ*ccp1* strains, resuspended in isotonic buffer, loaded with Mito-HyPer7 or Cyto-HyPer7, and exposed to 0.5 mM H_2_O_2_ for 30 min (“Treated”) or left untreated (“Control”). For the reduction control (panel *C*), protoplasts were incubated with 30 mM DTT for 10 min prior to measurement. Fluorescence excitation spectra (λ_em_ = 516 nm) were recorded from 360 to 506 nm. Panels (*C*–*E*) show mitochondrial HyPer7 responses; panels (*F* and *G*) show cytosolic HyPer7 responses. *H*, WT and Δ*ccp1* strains were treated with 0, 0.5, or 1 mM H_2_O_2_ for 30 min; for the “0.5 + 1” condition, cultures were primed with 0.5 mM H_2_O_2_ for 30 min and then exposed to 1 mM H_2_O_2_ for an additional 30 min mtDNA integrity was evaluated using long-short amplicon qPCR, and lesion frequency was calculated using the Poisson model (see Experimental procedures). n = 3 biological replicates. Experiments were repeated independently three times with similar results. *I*, WT and Δ*ccp1* strains were subjected to the same H_2_O_2_ treatment regimens as in panel h, followed by staining with 50 nM TMRM at 37 °C for 30 min. Red fluorescence indicates Δψm. For FCCP control, cells were pretreated with 10 μM FCCP for 30 min before TMRM staining. Scale bar, 20 μm. Images are representative of three independent biological replicates. *J*, Cytosolic GAPDH activity. WT and Δ*ccp1* strains were treated as in (*H*), harvested, and GAPDH activity was measured colorimetrically and normalized to the untreated control. *K*, Mitochondrial aconitase (mACO) activity. Strains were treated as in (*H*). Hyphae were harvested, and mACO activity was quantified and normalized to untreated controls. For (*H* and *K*), data represent mean ± SD, n = 3. *p* values shown were calculated by two-way ANOVA with Tukey’s multiple comparisons (∗∗∗∗*p* < 0.0001; ns, not significant).

To clarify how Ccp1 mediates oxidative adaptation, we first examined its localization and functional relevance. Confocal microscopy showed clear colocalization of Ccp1*–*GFP with Mito-Tracker Red before and after H_2_O_2_ treatment (Fig. 5*A*), indicating mitochondrial residence. Functionally, *gpdA* promoter (P*gpdA*) –driven overexpression of *ccp1* restored high H_2_O_2_ resistance in Δ*ccp1* even without priming, whereas removal of the mitochondrial targeting sequence (P*gpdA-ccp1∗*) completely abolished this effect (Figs. 5*B* and S2*E*), demonstrating that Ccp1 protection requires proper mitochondrial targeting.

Because yeast Ccp1 possesses both catalytic and redox-relay activities (32), we next examined whether its catalytic activity is required for oxidative adaptation in *A. nidulans*. Mutation of the conserved catalytic residue Trp191 (Ccp1^W191F^) eliminated H_2_O_2_ decomposition activity *in vitro* (Fig. S5, *A*–*C*) and failed to complement Δ*ccp1 in vivo* (Fig. 5*B*), indicating that Ccp1-mediated protection depends on its catalytic activity rather than a redox-relay mechanism.

To assess the impact of Ccp1 on mitochondrial redox homeostasis, we engineered a mitochondria-targeted HyPer7 probe (33) (Mito-HyPer7). Colocalization of Mito-HyPer7 with Mito-Tracker Red confirmed accurate mitochondrial targeting of the probe, whereas Cyto-HyPer7 fluorescence was restricted to the cytosol (Fig. S6). In Δ*ccp1*, Mito-HyPer7 exhibited a chronically oxidized pattern, which was fully reversible by 30 mM DTT (Fig. 5*C*, probe validation control), confirming the probe’s functionality in this strain. Under adaptive oxidative conditions, low-dose priming decreased Mito-HyPer7 oxidation in WT (Fig. 5*D*) but not in Δ*ccp1* (Fig. 5*E*), whereas cytosolic Cyto-HyPer7 responses were comparable between strains (Fig. 5, *F* and *G*), suggesting that redox imbalance is mitochondria specific.

To further characterize the mitochondrial defects, we assessed mtDNA integrity, membrane potential, and Fe-S enzyme activity. Low-dose priming protected mtDNA from oxidative damage in the wild type but not in Δ*ccp1* (Fig. 5*H*). TMRM imaging showed that primed wild-type hyphae retained strong mitochondrial membrane potential, whereas Δ*ccp1* remained depolarized under all conditions (Fig. 5*I*), indicating an essential role for Ccp1 in sustaining membrane potential. Consistently, mitochondrial aconitase activity was maintained in the wild type but sharply reduced in Δccp1 under challenge, while cytosolic GAPDH activity was unchanged (Fig. 5, *J* and *K*), confirming that the defects are mitochondria-specific.

Together, these results demonstrate that Ccp1-mediated adaptation relies on its sufficient expression, precise mitochondrial localization, and intact catalytic activity. By sustaining mitochondrial redox balance, membrane potential, and Fe-S enzyme integrity, Ccp1 prevents mtDNA damage and metabolic collapse, thereby acting as a key mitochondrial safeguard during oxidative adaptation.

### Mitochondrial defense alone is sufficient to sustain inducible antioxidant adaptation

**Figure 6:**
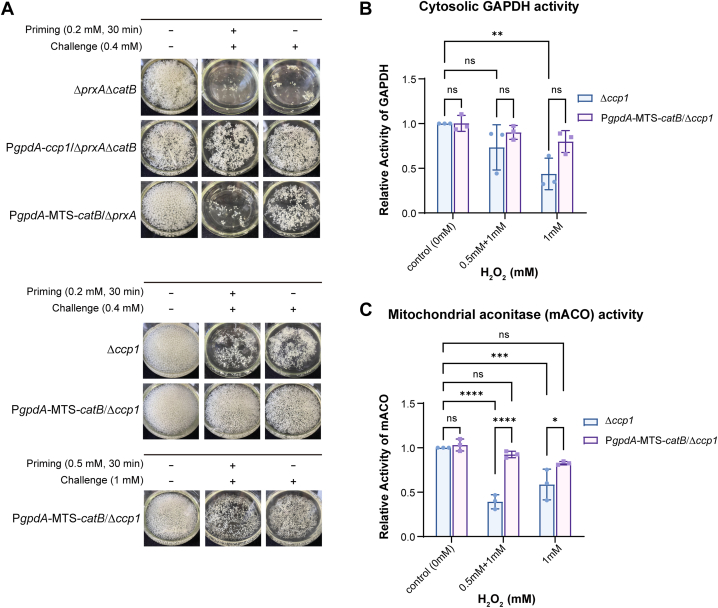
**Mitochondrial-targeted peroxidase activity sustains oxidative tolerance and reveals a “mitochondria-first” protection strategy.***A*, oxidative stress adaptation assays. (*top*) Δ*prxA*Δ*catB*, P*gpdA-ccp1*/Δ*prxA*Δ*catB*, P*gpdA*-MTS-*catB*/Δ*prxA*, and P*gpdA*-MTS-*catB*/Δ*ccp1* strains were grown for 4.5 h to the short-hypha stage and divided into control, primed, and unprimed groups. For the first three strains, priming was performed with 0.2 mM H_2_O_2_ for 30 min followed by challenge with 0.4 mM H_2_O_2_; for P*gpdA*-MTS-*catB*/Δ*ccp1*, priming used 0.5 mM H_2_O_2_ for 30 min followed by 1 mM H_2_O_2_. After a total of 12 h, oxidative tolerance was evaluated by hyphal pellet number and size. (*bottom*) Validation of priming intensity and tolerance in Δ*ccp1* and P*gpdA*-MTS-*catB*/Δ*ccp1* strains. Priming regimens of 0.5 → 1 mM or 0.2 → 0.4 mM H_2_O_2_ were compared. Quantification of mycelial dry weight corresponding to these phenotypes is shown in Figure S2F. *B*, cytosolic GAPDH (GpdA) activity. Δ*ccp1* and P*gpdA*-MTS-*catB*/Δ*ccp1* strains were treated with 0, 0.5, or 1 mM H_2_O_2_ for 30 min; the 0.5 mM group received an additional 1 mM H_2_O_2_ for 30 min. GAPDH activity was quantified colorimetrically and normalized to untreated controls. *C*, Mitochondrial aconitase (mACO) activity. Treatment conditions were identical to panel (*B*). Aconitase activity was measured colorimetrically and normalized to untreated samples. For (*B* and *C*), data represent mean ± SD (n = 3 biological replicates). Statistical significance is indicated as ns, ∗, ∗∗, ∗∗∗, ∗∗∗∗ (two-way ANOVA with Tukey’s multiple comparisons).

To test whether mitochondrial protection is not only necessary but also sufficient to establish adaptive oxidative resistance, we performed compensatory overexpression and mitochondrial-redirecting experiments in a highly H_2_O_2_-sensitive background lacking the major cytosolic and secretory peroxidases PrxA and CatB (Δ*prxA*Δ*catB*) (15). In this sensitized context, constitutive overexpression of mitochondrial Ccp1 restored strong H_2_O_2_ tolerance even without priming, whereas mild low-dose exposure caused slight growth inhibition rather than further improvement (Figs. 6*A*, top and S2*F*). These results indicate that enhancing mitochondrial peroxide removal can bypass canonical cytosolic detoxification pathways.

To further confirm sufficiency, we redirected CatB to mitochondria by replacing its secretion signal with a mitochondrial targeting sequence (P*gpdA-*MTS*-catB*). When expressed in the Δ*prxA* strain, MTS*-*CatB conferred robust resistance without priming (Figs. 6*A*, top and S2*F*), demonstrating that mitochondrial localization alone is adequate to confer antioxidant function. Moreover, introducing MTS*-*CatB into Δ*ccp1* similarly restored high-level resistance (Figs. 6*A*, bottom and S2*F*), showing that enhancing mitochondrial peroxide clearance can reconstitute adaptation even without Ccp1. Supporting this, in P*gpdA-*MTS*-catB*/Δ*ccp1*, cytosolic GAPDH activity remained stable (Fig. 6*B*) and mitochondrial aconitase activity was restored to wild-type levels (Fig. 6*C*), indicating recovery of mitochondrial redox and metabolic homeostasis.

Together, these results demonstrate that boosting mitochondrial H_2_O_2_ -scavenging capacity is sufficient to establish robust adaptive resistance independently of upstream signaling. Thus, antioxidant protection depends primarily on subcellular localization and catalytic competence, and mitochondrial reinforcement alone can sustain inducible oxidative adaptation.

## Discussion

**Figure 7:**
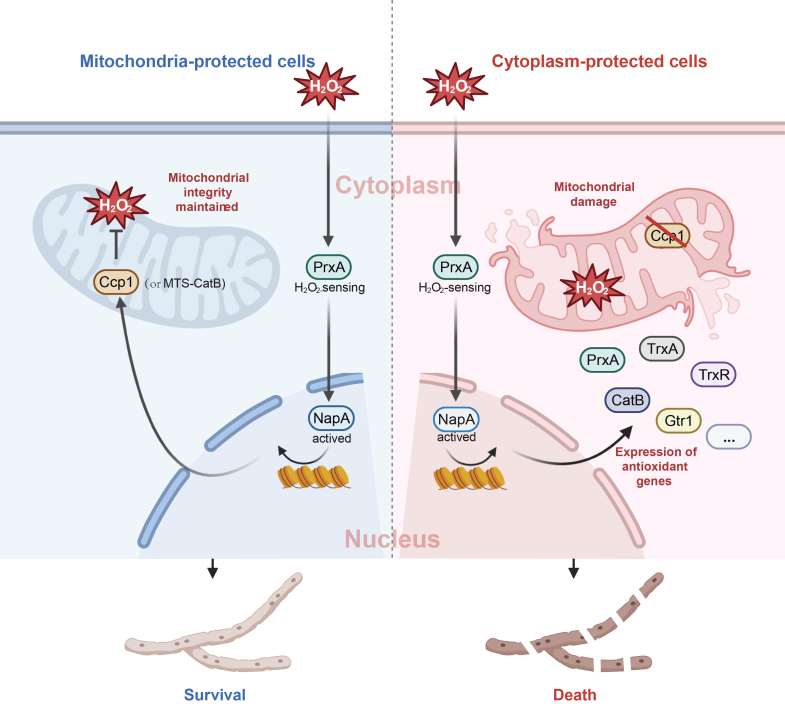
**Model illustrating the PrxA-NapA-Ccp1 axis-mediated mitochondrial protective mechanism underlying inducible oxidative adaptation.***Left* (mitochondria-protected cells): Low-dose H_2_O_2_ is sensed by cytosolic PrxA, which activates NapA to strongly induce the mitochondrial peroxidase Ccp1 and the artificially mitochondria-targeted MTS-CatB. Acting directly within mitochondria, these enzymes eliminate local ROS, preserve Δψm, protect Fe-S enzyme activity, and maintain mtDNA integrity, thereby enabling robust inducible oxidative tolerance even in the absence of cytosolic antioxidant systems. *Right* (cytoplasm-protected cells): In the absence of mitochondrial Ccp1, PrxA still activates NapA and induces cytosolic antioxidants such as CatB, TrxA, TrxR, and Gtr1. However, these enzymes cannot prevent rapid mitochondrial depolarization, mtDNA damage, or Fe-S enzyme inactivation under severe oxidative stress. Mitochondrial collapse destabilizes global metabolism and results in irreversible cell death. Overall principle: Oxidative adaptation is determined not by the abundance of antioxidant enzymes but by their precise mitochondrial localization. Enhancing mitochondrial H_2_O_2_ detoxification (*via* Ccp1 or MTS-CatB) alone is sufficient to restore adaptation, whereas strengthening cytosolic defenses is inadequate. This observation highlights the central role of maintaining mitochondrial integrity in oxidative adaptation.

This study defines the core logic of fungal oxidative adaptation and establishes a mitochondria-centered defensive framework, a “mitochondria-first protection” strategy. Upon low-dose H_2_O_2_ stimulation, PrxA acts primarily as a redox sensor rather than a detoxifying enzyme, transmitting oxidative cues to the transcription factor NapA. NapA activation triggers a focused antioxidant program, but its protective output converges almost exclusively on mitochondria through the induction of Ccp1. As summarized in our model (Fig. 7), when mitochondria receive priority protection—either through endogenous Ccp1 or engineered mitochondrial CatB—cells maintain Δψm, preserve Fe-S metabolism, and remain fully viable despite cytosolic vulnerability. By contrast, when Ccp1 is absent, cytoplasm-biased antioxidant defenses, even when strongly induced, fail to prevent mitochondrial collapse and ultimately cell death. Thus, oxidative resilience is governed not by the total amount of peroxide-scavenging enzymes, but by whether mitochondrial integrity is preserved, positioning the PrxA*–*NapA*–*Ccp1 axis as the decisive driver of adaptation.

In contrast to the classical Prx*–*Trx cycle model (30, 34, 35), our findings redefine the functional emphasis of PrxA in fungi. Although fungal PrxA has been viewed as a Trx-dependent cytosolic peroxidase, extensive work in mammalian and other animal systems shows that Prxs also act as key redox-signal transmitters (3, 5, 36). In *Schizosaccharomyces pombe*, Prx (Tpx1) relays H_2_O_2_ -derived signals to Pap1 through reversible oxidation (10, 37). Together with previous studies, our work demonstrates a similar PrxA→NapA redox relay in *Aspergillus* species (16, 19, 38). Because NapA also upregulates *prxA* expression, this pathway was long considered a feedback loop reinforcing PrxA-mediated detoxification. However, our genetic data reveal that PrxA’s principal role lies in oxidative-signal initiation and propagation. Unlike Δ*prxA*, the Δ*trxA* mutant showed enhanced resistance (Fig. 2*E*), indicating that PrxA’s signaling function—rather than its peroxidase activity—is the dominant contributor to antioxidant defense, whereas H_2_O_2_ removal represents a secondary role.

Transcriptomic and genetic analyses further identify Ccp1 as the major NapA-induced effector. Although yeast studies place Ccp1 within respiratory-chain peroxide detoxification (39), its broader role in filamentous fungi remained unclear. Here, we show that Ccp1 loss causes Δψm collapse, mtDNA damage, and loss of mitochondrial metabolic integrity; moreover, expressing MTS*-*CatB in the Δ*ccp1* background can also significantly restore H_2_O_2_ tolerance. These results highlight a fundamental principle: subcellular localization—not enzyme identity—determines antioxidant efficacy. Similar concepts appear in mammalian systems. Mitochondrial PRDX3 requires Prohibitin to maintain low mtROS and oxidative tolerance (40), and mitochondria-targeted antioxidants such as MitoQ reduce mtDNA damage *in vivo* (41). Although these systems differ mechanistically—transcriptional induction *versus* targeted enrichment—they converge on reinforcing mitochondrial defense as the decisive determinant of oxidative resilience, underscoring an evolutionarily conserved strategy of spatial prioritization.

Cells respond to low, non-damaging H_2_O_2_ because such cues predict impending oxidative stress and provide a preparatory window (1). The goal is to protect the components most vulnerable to irreversible damage. Structural and metabolic features point to mitochondria as the primary candidate: enriched Fe-S clusters, metal centers, high Δψm, and unshielded mtDNA collectively render them highly susceptible to depolarization, enzyme inactivation, and ROS amplification (42). Our measurements of Δψm, aconitase activity, and mtDNA integrity confirm that mitochondria are indeed the earliest and most sensitive points of oxidative failure. Notably, enhancing mitochondrial H_2_O_2_ clearance (*e.g.*, MTS*-*CatB) restores high-level resistance even in cytosolic peroxidase-deficient strains and without priming. Thus, low-dose H_2_O_2_ serves as a predictive signal enabling mitochondria fortification before lethal stress occurs. Therefore, Ccp1-mediated mitochondrial protective defense represents a key mechanism underlying oxidative tolerance in *A. nidulans*.

## Experimental procedures

### Strains and growth conditions

All *A. nidulans* strains used in this study are listed in Table S2. Unless otherwise noted, cultures were grown at 37 °C in minimal medium (MM) containing 1% (w/v) glucose, 10 mM NaNO_3_, 7 mM KCl, 10 mM KH_2_PO_4_, 2 mM MgSO_4_, and 2 ml/L Hunter’s trace elements (43), adjusted to pH 6.5; pyridoxine (0.4 mg/L), uracil (0.5 g/L), uridine (0.6 g/L), or biotin (0.4 mg/L) was added when required to satisfy auxotrophic markers. To characterize nitrogen-source–dependent regulation of the *niaD* promoter, reporter strains were grown in MM in which the sole nitrogen source was replaced with 70 mM NaNO_3_, 10 mM proline, or 5 mM ammonium tartrate. *E. coli* BL21(DE3) was used for recombinant protein expression and cultured in LB medium, whereas *E. coli* DH5α was used for plasmid propagation.

### Plasmid construction

All plasmids used in this study are listed in Table S3. Genomic DNA of *A. nidulans* strain A4 was extracted using a genomic DNA purification kit (Promega, Madison, WI, USA). Plasmids were constructed using the Hieff Clone Universal One-Step Cloning Kit (Yeasen, Shanghai, China), and PCR amplification was performed with 2 × Phanta Flash Master Mix DNA polymerase (Vazyme, Nanjing, China). Primer sequences are provided in Table S4. Basic backbone plasmids including pUC19-*pyrG*, pUC19-*pyroA*, pUC19-FLAG-T*trpC*-*pyroA*, and pUC19-*gfp*-T*trpC*-*pyroA* were obtained from the laboratory stock. To construct the *ccp1* expression plasmid pUC19-*pyroA*-P*gpdA*-*ccp1*-T*trpC*, the *gpdA* promoter, *E. coli ccp1* coding sequence, and *trpC* terminator were sequentially assembled downstream of the *pyroA* marker. P*gpdA* and T*trpC* fragments were amplified from the *A. nidulans* genome using PgpdA.F/PgpdA-ccp1.R and ccp1-TtrpC.F/TtrpC-pyroA.R, respectively, and the *ccp1* ORF was amplified using ccp1-orf.F/ccp1-orf.R. All fragments were inserted into linearized pUC19-*pyroA via* homologous recombination to generate the final plasmid. Point-mutation and signal-peptide-deleted variants were constructed using pUC19-*pyroA*-P*gpdA*-*ccp1*-T*trpC* as the template. The catalytic-inactive mutant pUC19-*pyroA*-P*gpdA*-*ccp1*^W191F^-T*trpC* was generated by reverse PCR with primers ccp1W191 F.F/ccp1W191 F.R to substitute Trp191. Deletion of the mitochondrial targeting sequence (MTS) was achieved using primers ccp1∗.F/gpdA-ccp1∗.R, followed by circularization in *E. coli* DH5α to obtain pUC19-*pyroA*-P*gpdA*-*ccp1*∗-T*trpC*. To construct the HyPer7 reporter plasmids, both mitochondrial-targeted and cytosolic versions of the probe were generated. The mitochondrial reporter plasmid pUC19-*pyroA*-P*gpdA*-MTS-*HyPer7*-T*trpC* was generated by fusing the mitochondrial targeting sequence (MTS) of Ccp1 to HyPer7. The backbone was linearized from pUC19-*pyroA*-P*gpdA*-*ccp1*-T*trpC* using TtrpC.F/ccp1-MTS.R to retain the MTS. The *HyPer7* fragment containing 20-bp homologous arms was amplified from pUC19-*pyrG*-P*gpdA*-*HyPer7*-T*trpC* using ccp1-HyPer7.F/HyPer7-TtrpC.R and assembled by one-step cloning. The cytosolic HyPer7 plasmid pUC19-*pyroA*-P*gpdA*-*HyPer7*-T*trpC* was derived from the mitochondrial construct by removing the MTS through reverse PCR using PgpdA-HyPer7.F/PgpdA-HyPer7.R.

### Construction of deletion strains

Deletion strains were generated using a CRISPR-Cas9-based strategy. sgRNAs were designed with CRISPRdirect (https://crispr.dbcls.jp/) and synthesized using the GeneArt Precision gRNA Synthesis Kit (Invitrogen). Primers used for assembling gRNA DNA templates and donor repair cassettes are listed in Tables S5 and S6. Previously established single-gene deletion strains included Δ*prxA*, Δ*catB*, Δ*prxA*Δ*catB*, Δ*gpx2*, Δ*napA*, Δ*tpsA*, and Δ*trxA* (15, 18, 19, 44). For construction of Δ*catA*, ∼1 kb 5′ and 3′ UTRs flanking *catA* were amplified from *A. nidulans* A4 genomic DNA (catA-up.F/catA-up.R; catA-down.F/catA-down.R) and used as homology arms, while the *pyrG* marker was amplified from pUC19-*pyrG* (pyrG.F/pyrG.R). These fragments were assembled into a donor cassette by fusion PCR (fu-catA.F/fu-catA.R), and a mixture containing 1 μg donor DNA, 3 μg sgRNA targeting *catA*, and 1 μg Cas9 protein was co-transformed into the wild type to obtain Δ*catA*. The same strategy was used to generate Δ*catC*, Δ*ccp1*, and Δ*atf1* in the wild-type background; Δ*ccp1*Δ*catB* and Δ*ccp1*Δ*catB*Δ*trxA* was constructed in the Δ*catB* and Δ*catB*Δ*trxA* background. To construct the Δ*catBΔtrxA* double mutant, the 5′ and 3′ homology arms of *catB* (catB-up.F/catB-up.R; catB-down.F/catB-down.R) and the *pyroA* marker (pyroA.F/pyroA.R) were amplified and assembled by fusion PCR (fu-catB.F/fu-catB.R), and the resulting donor cassette was co-transformed with sgRNA and Cas9 into the Δ*trxA* background. Using the same workflow, Δ*sodA*, and Δ*gedE* were generated, and Δ*napA*Δ*trxA* was constructed in the Δ*trxA* background. All transformants were initially screened by nutritional marker complementation and subsequently verified by colony PCR using the Direct DNA Amplification Kit (Jiangsu, China) and Sanger sequencing to confirm correct genotypes (Fig. S7; primers in Table S7).

### Construction of tagged strains

C-terminally tagged strains were generated to monitor endogenous protein localization and expression. The *catB-*FLAG strain was constructed previously (15). To generate the *ccp1-gfp* strain, the “*gfp::pyroA*” cassette was amplified from pUC19-*gfp*-T*trpC*-*pyroA* (pyroA.F/ccp1-gfp.R), while the *ccp1* ORF lacking the stop codon (ccp1-orf.F/ccp1-linker.R) and its 3′ UTR (pyroA-Tccp1.F/ccp1-down.R) were amplified from *A. nidulans* A4 genomic DNA as homology arms. The three fragments were assembled *via* fusion PCR (fu-ccp1-gfp.F/fu-ccp1-gfp.R) to construct the donor cassette (*ccp1-gfp-3′ccp1*), which was co-transformed with an sgRNA targeting the *ccp1* 3′ UTR and Cas9 into the wild type; positive transformants were identified by colony PCR (Fig. S8) and verified by Sanger sequencing (Kangwei Biotechnology). For construction of the *prxA-*FLAG strain, the *prxA* ORF retaining the start codon (prxA.F/prxA-FLAG.R) and its corresponding 3′ UTR (TprxA.F/prxA-down.R) were amplified from genomic DNA, while the “FLAG::*pyroA*” cassette was amplified from pUC19-FLAG-T*trpC*-*pyroA* (FLAG-TtrpC.F/pyroA-TprxA.R). These fragments were assembled by fusion PCR (fu-prxAFLAG.F/fu-prxAFLAG.R) to generate the donor cassette (*prxA-*FLAG::*pyroA-3′prxA*), which was co-transformed with the appropriate sgRNA and Cas9 into the wild type; correct integration was confirmed by colony PCR (Fig. S8) and sequencing.

### Construction of complementation and probe-expression strains

Complementation and probe-expression strains were generated as follows. The plasmids pUC19*-pyroA-*P*gpdA-ccp1-*T*trpC* (wild-type *ccp1*)*,* pUC19*-pyroA-*P*gpdA-ccp1∗-*T*trpC (*MTS-deleted *ccp1)*, and pUC19*-pyroA-*P*gpdA-ccp1*^W191F^*-*T*trpC* (catalytically inactive W191 F mutant) were individually transformed into the Δ*ccp1* strain, and transformants were selected *via pyroA* prototrophy to obtain the P*gpdA*-*ccp1*, P*gpdA*-*ccp1*∗, and P*gpdA*-*ccp1*^W191F^ strains. To monitor compartment-specific H_2_O_2_ dynamics, the mitochondrial-targeted HyPer7 construct (pUC19-pyroA-P*gpdA*-MTS-*HyPer7*-T*trpC*) and the cytosolic HyPer7 construct (pUC19-pyroA-P*gpdA*-*HyPer7*-T*trpC*) were transformed into both wild-type and Δ*ccp1* backgrounds, yielding the corresponding Mito-HyPer7 and Cyto-HyPer7 reporter strains.

### Construction of promoter-replacement and combinatorial-background strains

Promoter-replacement and combinatorial-background strains were generated as follows. In the Δ*prxA*Δ*catB* background, the *ccp1* 5′ homology arm and the P*gpdA-ccp1* fragment were amplified from genomic DNA and pUC19*-pyroA-*P*gpdA-ccp1-*T*trpC*, respectively (ccp1-up.F/ccp1-PgpdA.R; PgpdA.F/ccp1-orf.R), and assembled by fusion PCR (fu-Pccp1.F/fu-Pccp1.R) to generate the donor cassette for replacing the native *ccp1* promoter. This cassette was co-transformed with an sgRNA targeting the *ccp1* promoter region and Cas9, yielding the P*gpdA-ccp1*/Δ*prxA*Δ*catB* strain. Using the same strategy, P*gpdA-*MTS*-catB/*Δ*prxA* and P*gpdA*-MTS-*catB*/Δ*ccp1* strains were constructed in Δ*prxA* and Δ*ccp1* backgrounds, respectively. All transformants were verified by colony PCR (Figs. S9 and S10) and sequencing of key junctions. To generate conditional low-expression strains, the *trxR* 5′ UTR homology arm, the *niaD* promoter, the *trxR* 3′ UTR arm, and the *pyroA* selectable marker were amplified from genomic DNA and pUC19-*pyroA* (trxR-up.F/trxR-pyroA.R; PniaD.F/PniaD.R; PniaD-trxR.F/trxR-down.R; pyroA.F/pyroA-PniaD.R) and assembled by fusion PCR (fu-trxR.F/fu-trxR.R) to construct the donor cassette for replacing the native *trxR* promoter. Co-transformation with the sgRNA targeting the *trxR* promoter and Cas9 yielded the P*niaD*-*trxR* strain. Using the same promoter-replacement strategy, P*niaD*-*gtr1* and P*niaD*-*grx5* strains were constructed in the wild-type background; the P*niaD-prxA-*FLAG strain was constructed in the *prxA-*FLAG background. All transformants were confirmed by colony PCR (Fig. S11) and sequencing of critical regions.

### H_2_O_2_ dose–response analysis and LC50 determination

Conidia were harvested from 48-h cultures of the WT, Δ*prxA*, Δ*napA*, and Δ*ccp1* strains and resuspended in sterile saline to a final concentration of 1 × 10^6^ spores/ml. The conidial suspensions were inoculated into 100 ml minimal medium (MM) and incubated at 37 °C with shaking at 220 rpm for 4.5 h. Hydrogen peroxide was then added to final concentrations of 0, 0.05, 0.1, 0.2, 0.3, 0.4, 0.6, 0.8, 1.0, 1.2, and 1.5 mM. After an additional 12 h incubation, mycelia were harvested and the dry weight was measured to evaluate fungal growth under oxidative stress. Mycelial dry weight from each treatment was normalized to the untreated control, and dose–response curves were generated accordingly. LC50 values (the H_2_O_2_ concentration causing 50% growth inhibition) were calculated by nonlinear regression analysis using GraphPad Prism. All experiments were performed with at least three independent biological replicates.

### Oxidative stress adaptation assays

Conidia of *A. nidulans* were harvested from 48-h cultures using sterile saline and resuspended to a final concentration of 1 × 10^6^ spores/ml, followed by inoculation into 100 ml minimal medium (MM) and shaking incubation at 37 °C and 220 rpm for 4.5 h. For the pre-treatment regimen, cultures were first exposed to a low concentration of H_2_O_2_ (0.2 or 0.5 mM) for 30 min and subsequently challenged with a higher dose (0.5 or 1 mM). In the no-pre-treatment group, the high-dose H_2_O_2_ (0.5 or 1 mM) was added directly after 4.5 h of growth, whereas the control group received no H_2_O_2_. All cultures were then incubated for an additional 12 h at 37 °C and 220 rpm. After treatment, equal culture volumes were collected for phenotypic and quantitative analyses, including hyphal morphology, cell viability, and biomass determination. Hyphal pellet size and abundance were examined as indicators of H_2_O_2_ sensitivity. Cell viability was assessed using the WST-1 Cell Proliferation and Cytotoxicity Assay (Beyotime, Shanghai, China). Briefly, 1 × 10^5^ conidia were inoculated into 96-well plates containing 200 μl MM per well and incubated at 37 °C for 4.5 h; the low-dose and pre-treatment groups were exposed to 0.5 mM H_2_O_2_ for 30 min, after which 1 mM H_2_O_2_ and the WST-1 reagent were added to the pre-treatment and no-pre-treatment groups simultaneously, followed by incubation at 37 °C for 5 h. Absorbance at 450 nm (OD_450_) was measured using a microplate reader (Bio-Rad Model 550), and cell viability was calculated as: viability (%) = (mean OD of treatment/mean OD of untreated control) × 100%. For dry biomass determination, equal culture volumes were filtered after 12 h of treatment, washed thoroughly with distilled water, dried at 80 °C to constant weight, and weighed to obtain dry biomass (mg/ml). All assays were performed with at least three independent biological replicates, and data are presented as mean ± SD. Statistical significance was evaluated using one-way ANOVA.

### Construction, expression, and purification of recombinant Ccp1

The coding sequence of the cytochrome *c* peroxidase gene (*ccp1*) (with the signal peptide region removed) from *A. nidulans* was amplified from cDNA using primers ccp1-SUMO.F/ccp1-SUMO.R (Table S4). The PCR product was digested with BamHI and NotI and inserted into the pET28a*-*SUMO expression vector to generate pET28a*-*SUMO*-ccp1* after transformation into *E. coli* DH5α. A catalytic-site mutant construct, pET28a*-*SUMO*-ccp1*^W191F^, was produced by reverse PCR using primers ccp1W191 F.F/ccp1W191 F.R. After sequence verification, recombinant plasmids were transformed into *E. coli* BL21(DE3) competent cells for protein expression. Transformants were cultured in LB medium containing 50 μg/ml kanamycin at 37 °C until OD_600_ reached ∼0.6, followed by induction with 0.2 mM IPTG and incubation at 30 °C for 6 h. Cells were collected by centrifugation (8000×*g*, 10 min, 4 °C), resuspended in PBS, and subjected to protein purification. Recombinant proteins were purified using a HisTrap FF affinity column (GE Healthcare) according to the manufacturer’s instructions. After binding of clarified lysates, proteins were eluted stepwise with buffers containing 20 mM, 50 mM, and 250 mM imidazole. Eluted fractions were pooled, dialyzed to remove imidazole, and analyzed by SDS*–*PAGE, which confirmed a major band of ∼49.2 kDa. Protein concentrations were quantified using the Bradford assay, and purified proteins were stored at −80 °C until use.

### Ccp1 and Ccp1W191F enzyme activity assays

For *in vitro* characterization of recombinant Ccp1 and its catalytic mutant Ccp1^W191F^, purified proteins were subjected to kinetic analysis to determine the contribution of residue Trp191 to enzymatic activity. Reactions were performed in a total volume of 1 ml containing 50 mM PBS (pH 7.0), 40 μM reduced cytochrome *c* (generated by ascorbate reduction; Solarbio), 8 μg purified Ccp1 or Ccp1^W191F^, and various concentrations of H_2_O_2_ (10*–*400 μM). The decrease in absorbance at 550 nm was monitored in real time at 25 °C using a UV-5100 spectrophotometer (Hitachi, Japan). The initial reaction rate (V_0_) was calculated by measuring the change in absorbance, with values expressed as μM/min/mg protein. All enzymatic assays were performed with at least three independent biological replicates, and data are reported as mean ± SD; statistical significance was assessed using two-way ANOVA.

### Assessment of mitochondrial DNA integrity and membrane potential

Mitochondrial DNA integrity was assessed using a long-short amplicon qPCR assay targeting the mitochondrial *cox1* gene (45). Conidia were inoculated at 1 × 10^6^ spores/ml and grown for 5 h before exposure to the indicated H_2_O_2_ treatments. Total DNA was extracted using the Wizard Genomic DNA Purification Kit (Promega), quantified, and diluted to 16 ng/μl for qPCR analysis. Two amplicons were used: a short fragment (∼200 bp), serving as an amplification control for normalization; a semi-long fragment (∼2 kb), used to quantify DNA damage because lesions impede polymerase progression. qPCR was performed on a Bio-Rad CFX-96 system using equal amounts of DNA template for all reactions. Lesion frequency per 10 kb of DNA was calculated according to a Poisson-based model using the relative amplification of the semi-long fragment compared with the short fragment. The number of lesions per 10 kb DNA was calculated as:

$\text{Lesions}\;\text{per}\;10\;\text{kb}\;\text{DNA}=\frac{-\ln\left(A_{t}/A_{0}\right)\;\times \;10,000}{\text{size}\;\text{of}\;\text{semi}-\text{long}\;\text{fragment}\;\left(\text{bp}\right)}$ where A_t_ is the amplification quantity of the treated sample and A_0_ is that of the untreated control.

Mitochondrial membrane potential (Δψm) was assessed using the potentiometric dye TMRM (MCE HY-D0984). Hyphae grown on confocal dishes for 4.5 h were treated with 0, 0.5, or 1 mM H_2_O_2_ for 30 min; in pre-treated samples, 0.5 mM H_2_O_2_ was followed by 1 mM for an additional 30 min. After treatment, hyphae were washed twice with PBS and incubated with 50 nM TMRM for 30 min at room temperature. FCCP (10 μM, 30 min) was used as the depolarization control. Fluorescence was measured at Ex 561 nm/Em 595 nm, and Δψm was quantified from TMRM fluorescence intensity. All assays were performed using at least three biological replicates.

### Protein detection and immunoblotting

To examine how oxidative stress and nitrogen-source conditions affect the abundance of PrxA, CatB, and Ccp1, immunoblotting was performed using *A. nidulans* strains expressing PrxA-FLAG, CatB-FLAG, or Ccp1-GFP. Strains were grown in liquid MM at 37 °C with shaking for 4.5 h to the short-hypha stage and subjected to either H_2_O_2_ treatment or nitrogen-source modulation. For oxidative-stress assays, cultures were divided into control, low-dose, pretreated, and non-pretreated groups; the low-dose and pretreated groups were first exposed to 0.5 mM H_2_O_2_ for 30 min, after which the pretreated and non-pretreated groups received 1 mM H_2_O_2_ for an additional 30 min. For nitrogen-source assays, PrxA-FLAG strains driven by the *niaD* promoter were cultured for 5 h in MM containing 10 mM NaNO_3_, 10 mM proline, or 5 mM ammonium tartrate as the sole nitrogen source. After treatment, mycelia or culture supernatants were harvested. Mycelial samples were frozen and ground in liquid nitrogen, followed by lysis in ice-cold PBS to extract total protein. To preserve the *in vivo* redox state of PrxA, 25 mM N-ethylmaleimide (NEM) was added to the protein extracts immediately after cell disruption to alkylate free thiols and prevent post-lysis oxidation. Protein samples were prepared using either non-reducing or reducing SDS loading buffer and analyzed by SDS–PAGE. Extracellular CatB-FLAG was collected by filtering supernatants to remove biomass and concentrating the filtrate using ultrafiltration tubes (Millipore). For the detection of the mitochondrial protein Ccp1-GFP, frozen mycelia were ground in liquid nitrogen and lysed in RIPA buffer on ice for 30 min. Lysates were clarified by centrifugation (4 °C, 500×*g*, 10 min), briefly sonicated on ice, and centrifuged again (11,000×*g*, 2 min) to obtain total protein extracts. Protein concentrations were determined by Bradford or BCA assays and normalized to 1 mg/ml; 20 μg of protein was loaded per lane. Samples were resolved by SDS–PAGE (15% gels for PrxA-FLAG; 10% gels for Ccp1-GFP and CatB-FLAG) and transferred to PVDF membranes (Millipore). Membranes were blocked with 5% skim milk for 1 h and probed with mouse anti-FLAG monoclonal antibody (TransGen Biotech) or rabbit anti-GFP antibody (Abcam), followed by HRP-conjugated goat anti-mouse or anti-rabbit secondary antibodies (TransGen Biotech). To verify equal loading, membranes were reprobed with anti-GAPDH antibody (Sigma-Aldrich) after detection of target proteins. Signals were developed using ECL reagents (Tanon), imaged on a ChemiDoc system (Bio-Rad), and quantified by densitometry using ImageJ (NIH, USA). All assays were performed with at least three independent biological replicates.

### Quantitative real-time PCR (qRT-PCR)

Total RNA was extracted from each *A. nidulans* strain using the HiPure Total RNA Plus Kit (Magen), and first-strand cDNA was synthesized with the Hifair AdvanceFast first Strand cDNA Synthesis Kit (No Dye) (Yeasen). qRT-PCR was performed on a CFX-96 real-time PCR system (Bio-Rad) using SYBR Green PCR Master Mix (Bioligo). Gene-specific primers targeting oxidative stress–related genes (*prxA*, *trxR*, *ccp1*, *catB*, *trxA*, *sodA*, *gedE*, *gtr1*, *grx5*, *catC*, *gpx2*, *catA*, *tpsA*, *atf1*, *napA*) and the internal control *actin* were used (primer sequences listed in Table S8). All reactions included technical replicates, and each strain was analyzed with at least three independent biological replicates. Relative transcript abundance was calculated using the 2^−ΔΔCt^ method with normalization to *actin* expression. Data are presented as mean ± SD, and statistical significance was assessed using one-way ANOVA followed by Dunnett’s *post hoc* test, with significance thresholds set at *p* < 0.05, *p* < 0.01, *p* < 0.001, and *p* < 0.0001.

### Fluorescence microscopy and mitochondrial localization

To visualize the subcellular localization of Ccp1 and the associated redox probes, approximately 1 × 10^5^ conidia were suspended in 200 μl of minimal medium (MM) and inoculated into 35-mm confocal dishes, followed by incubation at 37 °C for 10 h to allow short hyphae to develop. Samples were gently washed twice with prewarmed PBS (pH 7.4) before imaging. Fluorescence imaging was performed using a Leica TCS SP8 confocal laser-scanning microscope (Leica). For strains expressing Ccp1-GFP, excitation was set to 488 nm and emission was collected at 525 nm. For mitochondrial staining, hyphae were incubated with 100 nM Mito-Tracker Red CMXRos (Yeasen, Shanghai) at 37 °C for 10 min in the dark, washed three times with PBS, and imaged immediately; excitation and emission wavelengths were 561 nm and 595 nm, respectively. Mitochondrial localization of Ccp1 was assessed by overlap of GFP and Mito-Tracker Red signals. The same imaging procedures were applied to strains expressing cytosolic or mitochondria-targeted HyPer7 probes (Cyto-HyPer7 and Mito-HyPer7).

### Quantitative analysis of cytosolic and mitochondrial H_2_O_2_ levels in protoplasts

To quantitatively monitor dynamic changes in H_2_O_2_ levels within the cytosol and mitochondria, engineered strains expressing cytosolic or mitochondria-targeted HyPer7 probes (Cyto-HyPer7 and Mito-HyPer7; construction described in Section 2.3) were employed. Measurements were performed using a protoplast-based assay to ensure compartment specificity and quantitative accuracy. Fluorescence was recorded on a spectrofluorometer (F-7000, Hitachi, Japan) with excitation at 400 nm and 499 nm and emission at 516 nm, using a 10-nm slit width. Wild-type and Δ*ccp1* strains expressing Cyto-HyPer7 or Mito-HyPer7 were cultured for 5 h under standard conditions, converted into protoplasts, and adjusted to identical concentrations. Samples were treated with 0.5 mM H_2_O_2_ for 30 min to induce oxidative stress, followed by the addition of 30 mM DTT to verify probe reversibility. All experiments were performed with at least three independent biological replicates, and results are presented as mean ± SD.

### Measurement of intracellular H_2_O_2_ in hyphae using BES-H_2_O_2_-Ac

Intracellular H_2_O_2_ levels were quantified using the selective fluorescent probe BES-H_2_O_2_-Ac (Wako). Short hyphae cultured at 37 °C for ∼4.5 h were incubated with the probe for 2 h to allow intracellular loading, then exposed to 0, 0.5, or 1 mM H_2_O_2_. Fluorescence intensity was recorded every 30 min using a spectrofluorometer (F-4600, Hitachi, Japan) with excitation at 485 nm and emission at 515 nm. Time-dependent fluorescence changes reflected intracellular H_2_O_2_ accumulation and clearance dynamics. All experiments were performed with three independent biological replicates, and results are expressed as mean ± SD.

### Measurement of cellular H_2_O_2_ degradation capacity

Extracellular H_2_O_2_ clearance assay was performed using hyphae grown in MM for 4.5 h at 37 °C to the short-hyphal stage. For priming, cultures were treated with 0.5 mM H_2_O_2_ for 30 min before the addition of 1 mM H_2_O_2_ to initiate the clearance assay. At the onset of treatment, 1 ml culture supernatants were collected every 30 min and stored at −20 °C H_2_O_2_ concentrations were quantified using a Hydrogen Peroxide Assay Kit (Beyotime S0038), in which H_2_O_2_ oxidizes Fe^2+^ to Fe^3+^ that forms a purple complex with xylenol orange. Samples were incubated with working solution for 30 min at room temperature, absorbance was measured at 560 nm, and H_2_O_2_ concentrations were calculated against a standard curve. Enzymatic H_2_O_2_ degradation in cell extracts was assessed by harvesting hyphae after the indicated treatments, grinding them in liquid nitrogen, and extracting proteins in pre-chilled PBS (pH 7.4). Clarified lysates were normalized by protein concentration using the Bradford assay. Peroxidase-dependent degradation of H_2_O_2_ was measured using the Amplex Red Hydrogen Peroxide/Peroxidase Assay Kit (Thermo Fisher Scientific A22188). Equal volumes of extract and Amplex Red/HRP working solution were mixed in black 96-well plates, and fluorescence was recorded at Ex 530 to 560 nm/Em 590 nm over 0 to 60 s to derive degradation rates. GAPDH and aconitase activities (Solarbio BC2210 and BC4480) were quantified as cytosolic and mitochondrial enzymatic controls, and all activities were normalized to total protein.

### Gene expression profiling

For transcriptomic analysis, fresh conidia of the wild-type strain were inoculated into liquid medium and cultured for 4.5 h. The culture was then divided equally into three flasks and treated with 0, 0.5, or 1 mM H_2_O_2_ for 15 min. Three independent biological replicates were used for each condition. Mycelia were rapidly harvested by centrifugation, flash-frozen in liquid nitrogen, and stored at −80 °C until RNA extraction. RNA extraction, library construction, sequencing, and downstream analyses were performed by Genedenovo Biotechnology Co. Total RNA was isolated using the TRIzol reagent (Invitrogen) following the manufacturer’s protocol. RNA quality and integrity were assessed using an Agilent 2100 Bioanalyzer (Agilent Technologies) and confirmed by RNase-free agarose gel electrophoresis. Eukaryotic mRNA was enriched using Oligo (dT) magnetic beads to remove rRNA. The enriched mRNA was fragmented in fragmentation buffer and reverse-transcribed to generate cDNA. After purification, double-stranded cDNA fragments were end-repaired and adenylated, and sequencing libraries were constructed. Libraries were sequenced on the Illumina NovaSeq 6000 platform by Genedenovo. Bioinformatic analyses were performed using the Omicsmart platform (https://www.omicsmart.com/). Gene expression levels were normalized as FPKM (Fragments Per Kilobase of transcript per Million mapped reads). Differentially expressed genes were identified using DESeq2 with the thresholds false discovery rate (FDR) < 0.05 and |fold change| ≥ 2.

### Statistical analysis

All statistical analyses were performed using GraphPad Prism v.9.5.0. All experiments were performed with at least three independent biological replicates. A biological replicate represents an independent culture initiated from a separate inoculum and processed independently. Quantitative data were derived from independent cultures rather than technical repeats. Data are presented as mean ± standard deviation (SD). Statistical significance was evaluated using one-way or two-way analysis of variance (ANOVA), as appropriate, followed by Tukey or Dunnett multiple-comparison tests. The specific statistical tests used for each experiment are indicated in the corresponding figure legends. Differences were considered statistically significant at *p* < 0.05.

## Data and code availability

The RNA-sequencing data generated in this study have been deposited in the NCBI Sequence Read Archive (SRA) under BioProject ID PRJNA1372280 (SRA Study accession: SRP650475) and will be made publicly available upon publication.

## Supporting information

This article contains supporting information (15, 18, 19, 44).

## Conflict of interest

The authors declare that they have no conflicts of interest with the contents of this article.

## Supporting information

Revised Supplemental_Information_Strains_and_Primers
