# Supplementary material for: Fungal oxidative stress tolerance depends on peroxiredoxin PrxA-mediated redox signaling to mitochondrial cytochrome c peroxidase Ccp1
Source: J Biol Chem. 2026 Apr 27;302(6):113084. doi: 10.1016/j.jbc.2026.113084 (PMC13253075; doi:10.1016/j.jbc.2026.113084)
Supplement: Revised Supplemental_Information_Strains_and_Primers [file mmc1.docx]

**Supplemental Information**

**Table of contents:**

**Table S2 Strains used in this study 2**

**Table S3 Plasmids used in this study 5**

**Table S4 Primers used for plasmids construction 6**

**Table S5 Primers used for sgRNA synthesis 7**

**Table S6 Primers used for strains construction 8**

**Table S7 Primers used for strains verification 14**

**Table S8 Primers used for qRT-PCR 16**

**Supplementary References 17**

**Table S2 Strains used in this study**

| **Strains** | **Genotype** | **Source** |
| --- | --- | --- |
| WT | *biA1;argB2;pyrG89;wA3;pyroA4* | Our Lab |
| Δ*prxA* | *biA1;argB2;pyrG89;wA3;pyroA4;* Δ*prxA::argB* | Our Lab ^1^ |
| Δ*catB* | *biA1;argB2;pyrG89;wA3;pyroA4;* Δ*catB::argB* | Our Lab ^2^ |
| Δ*catB*Δ*prxA* | *biA1;argB2;pyrG89;wA3;pyroA4;*  Δ*prxA::argB;*Δ*catB::pyrG* | Our Lab ^3^ |
| Δ*gpx2* | *biA1;argB2;pyrG89;wA3;pyroA4;* Δ*gpx2::argB* | Our Lab ^4^ |
| Δ*trxA* | *biA1;argB2;pyrG89;wA3;pyroA4;* Δ*trxA::argB* | Our Lab ^4^ |
| Δ*napA* | *biA1;argB2;pyrG89;wA3;pyroA4;* Δ*napA::argB* | Our Lab ^4^ |
| Δ*tpsA* | *biA1;argB2;pyrG89;wA3;pyroA4;* Δ*tpsA::pyrG* | Our Lab (unpublished) |
| *catB*-FLAG | *biA1; argB2;pyrG89;wA3;pyroA4;*  Δ*catB::catB:*FLAG*:pyrG* | Our Lab ^3^ |
| *prxA*^C31S^ | *biA1;argB2;pyrG89;wA3;pyroA4;*  Δ*prxA::prxA*^C31S^ | Our Lab ^4^ |
| *prxA*^C61S^ | *biA1;argB2;pyrG89;wA3;pyroA4;*  Δ*prxA::prxA*^C61S^ | Our Lab ^4^ |
| *prxA*^C61S/C31S^ | *biA1;argB2;pyrG89;wA3;pyroA4;*  Δ*prxA::prxA*^C61S/C31S^ | Our Lab ^4^ |
| Δ*catA* | *biA1;argB2;pyrG89;wA3;pyroA4;*  Δ*catA::pyrG* | This paper |
| Δ*catC* | *biA1;argB2;pyrG89;wA3;pyroA4;*  Δ*catC::pyrG* | This paper |
| Δ*napA*Δ*trxA* | *biA1;argB2;pyrG89;wA3;pyroA4;*  Δ*trxA::argB;*Δ*napA::pyroA* | This paper |
| Δ*catB*Δ*trxA* | *biA1;argB2;pyrG89;wA3;pyroA4;*  Δ*trxA::argB;*Δ*catB::pyroA* | This paper |
| Δ*sodA* | *biA1;argB2;pyrG89;wA3;pyroA4;*  Δ*sodA::pyroA* | This paper |
| Δ*gedE* | *biA1;argB2;pyrG89;wA3;pyroA4;*  Δ*gedE::pyroA* | This paper |
| Δ*atf1* | *biA1;argB2;pyrG89;wA3;pyroA4;*  Δ*atf1::pyrG* | This paper |
| P*niaD-trxR* | *biA1;argB2;pyrG89;wA3;pyroA4;*  ΔP*trxR::pyroA:*P*niaD* | This paper |
| P*niaD-gtr1* | *biA1;argB2;pyrG89;wA3;pyroA4;*  ΔP*gtr1::pyrG:*P*niaD* | This paper |
| P*niaD-grx5* | *biA1;argB2;pyrG89;wA3;pyroA4;*  ΔP*grx5::pyrG:*P*niaD* | This paper |
| P*niaD-prxA-*FLAG | *biA1; argB2;pyrG89;wA3;pyroA4;*  ΔP*prxA::pyrG:*P*niaD;*Δ*prxA::prxA:*FLAG*:pyroA* | This paper |
| Δ*ccp1* | *biA1;argB2;pyrG89;wA3;pyroA4;*  Δ*ccp1::pyrG* | This paper |
| Δ*ccp1*Δ*catB* | *biA1;argB2;pyrG89;wA3;pyroA4;*  Δ*catB::argB;*Δ*ccp1::pyrG* | This paper |
| Δ*ccp1*Δ*catB*Δ*trxA* | *biA1;argB2;pyrG89;wA3;pyroA4;*  Δ*trxA::argB;*Δ*catB::pyroA;*Δ*ccp1::pyrG* | This paper |
| *prxA-*FLAG | *biA1;argB2;pyrG89;wA3;pyroA4;*  Δ*prxA::prxA:*FLAG*:pyroA* | This paper |
| *ccp-gfp* | *biA1;argB2;pyrG89;wA3;pyroA4;*  Δ*ccp1::ccp1:gfp:pyroA* | This paper |
| P*gpdA*-MTS-*HyPer7*/WT | *biA1;argB2;pyrG89;wA3;pyroA4;*  Δ*pyroA::*P*gpdA*-MTS-*HyPer7:pyroA* | This paper |
| P*gpdA*-MTS-*HyPer7*/Δ*ccp1* | *biA1;argB2;pyrG89;wA3;pyroA4;*  Δ*ccp1::pyrG;*Δ*pyroA::*P*gpdA*-MTS-*HyPer7:pyroA* | This paper |
| P*gpdA*-*HyPer7*/WT | *biA1;argB2;pyrG89;wA3;pyroA4;*  Δ*pyroA::*P*gpdA*-*HyPer7:pyroA* | This paper |
| P*gpdA*-*HyPer7*/Δ*ccp1* | *biA1;argB2;pyrG89;wA3;pyroA4;*  Δ*ccp1::pyrG;*Δ*pyroA::*P*gpdA*-*HyPer7:pyroA* | This paper |
| P*gpdA*-*ccp1*/∆*ccp1* | *biA1;argB2;pyrG89;wA3;pyroA4;*  Δ*ccp1::pyrG;*Δ*pyroA::*P*gpdA*-*ccp1:pyroA* | This paper |
| P*gpdA*-*ccp1*^W191F^/∆*ccp1* | *biA1;argB2;pyrG89;wA3;pyroA4;*  Δ*ccp1::pyrG;*Δ*pyroA::*P*gpdA*-*ccp1*^W191F^*:pyroA* | This paper |
| P*gpdA*-*ccp1**/∆*ccp1* | *biA1;argB2;pyrG89;wA3;pyroA4;*  Δ*ccp1::pyrG;*Δ*pyroA::*P*gpdA*-*ccp1*:pyroA* | This paper |
| P*gpdA-ccp1*/Δ*prxA*Δ*catB* | *biA1; argB2;pyrG89;wA3;pyroA4;*  Δ*prxA::argB;*Δ*catB::pyrG;*ΔP*ccp1::pyroA:*P*gpdA* | This paper |
| P*gpdA-*MTS*-catB*/Δ*prxA* | *biA1; argB2;pyrG89;wA3;pyroA4;* Δ*prxA::argB;*ΔP*catB::pyroA:*P*gpdA:MTS:catB* | This paper |
| P*gpdA-*MTS*-catB*/Δ*ccp1* | *biA1; argB2;pyrG89;wA3;pyroA4;* Δ*ccp1::pyrG;*ΔP*catB::pyroA:*P*gpdA:MTS:catB* | This paper |

*ccp*: ccp* lack of N-terminal signal peptides.

MTS: Mitochondrial Targeting Signal of Ccp1.

**Table S3 Plasmids used in this study**

| **Plasmid** | **Source** |
| --- | --- |
| pUC19-*pyrG* | Our Lab |
| pUC19-*pyroA* | Our Lab |
| pUC19-*gfp*-T*trpC*-*pyroA* | Our Lab |
| pUC19-FLAG-T*trpC*-*pyroA* | Our Lab |
| pUC19-*pyrG*-P*gpdA*-*HyPer7*-T*trpC* | Our Lab |
| pET28a-SUMO | Our Lab |
| pUC19-*pyroA*-P*gpdA*-*ccp1*-T*trpC* | This paper |
| pUC19-*pyroA*-P*gpdA*-*ccp1*^W191F^-T*trpC* | This paper |
| pUC19-*pyroA*-P*gpdA*-*ccp1**-T*trpC* | This paper |
| pUC19-*pyroA*-P*gpdA*-*MTS-HyPer7*-T*trpC* | This paper |
| pUC19-*pyroA*-P*gpdA*-*HyPer7*-T*trpC* | This paper |
| pET28a-SUMO-*ccp1* | This paper |
| pET28a-SUMO-*ccp1*^W191F^ | This paper |

**Table S4 Primers used for plasmids construction**

| **Primers** | **Template** | **Nucleotide sequence (5’ to 3’)** |
| --- | --- | --- |
| PgpdA.F | FGSC A4 | GCTCCTTATTGAAGTCGGAG |
| PgpdA-ccp1.R |  | GATCGAGCAGCGGAAGCCATTGTGATGTCTGCTCAAGCGG |
| ccp1-orf.F | FGSC A4 | ATGGCTTCCGCTGCTCGATC |
| ccp1-orf.R |  | TTACTCAGACCTCTTGAAGAC |
| ccp1-TtrpC.F | FGSC A4 | GTCTTCAAGAGGTCTGAGTAATAGTGATTTAATAGCTCC |
| TtrpC-pyroA.R |  | CTCCTGGATCCGAATTCGAGCTCGAAAGAAGGATTACCTCTAAACAAGTG |
| ccp1W191F.F | pUC19-*pyroA-*P*gpdA-ccp1-*T*trpC* | GATGGACCCTTCAACTTCAGCCCTACCGTC |
| ccp1W191F.R |  | GGGCTGAAGTTGAAGGGTCCATCGAAACCAGAG |
| ccp1*.F | FGSC A4 | TCTTCCGAGGCGAGCTCC |
| gpdA-ccp1*.R |  | GGAGCTCGCCTCGGAAGACATTGTGATGTCTGCTCAAGCGG |
| TtrpC.F | pUC19-*pyroA-*P*gpdA-ccp1-*T*trpC* | ATAGTGATTTAATAGCTCCATG |
| ccp1-MTS.R |  | GTATCCACGGCGAGAAGCAAC |
| ccp1-Hyper7.F | pUC19-*pyrG-*P*gpdA*-*HyPer7*-T*trpC* | GTTGCTTCTCGCCGTGGATACATGCACCTCGCCAACGAGG |
| Hyper7-TtrpC.R |  | CATGGAGCTATTAAATCACTATTAGTCGCAGATGAAGGAG |
| PgpdA-Hyper7.F | pUC19-*pyroA-*P*gpdA-*MTS-*HyPer7*-T*trpC* | GCTTGAGCAGACATCACAATGCACCTCGCCAACGAGG |
| PgpdA-Hyper7.R |  | CATTGTGATGTCTGCTCAAGCGGG |
| ccp1-SUMO.F | cDNA | GAGAACAGATTGGTGGATCCTCTTCCGAGGCGAGCTC |
| ccp1-SUMO.R |  | GGTGCTCGAGTGCGGCCGCTTACTCAGACCTCTTGAAGAC |

**Table S5 Primers used for sgRNA synthesis**

| **Primers** | **Gene location** | **Nucleotide sequence (5’ to 3’)** |
| --- | --- | --- |
| *catA*-sgF | *catA* | TAATACGACTCACTATAGGACCTATGCTGGAGTG |
| *catA*-sgR |  | TTCTAGCTCTAAAACTTAACACTCCAGCATAGGTC |
| *catB*-sgF | *catB* | TAATACGACTCACTATAGAGGAGGACCAGTTGCGCTT |
| *catB*-sgR |  | TTCTAGCTCTAAAACAAGCGCAACTGGTCCTCCTC |
| *catC*-sgF | *catC* | TAATACGACTCACTATAGCACCGTCATCGACATG |
| *catC*-sgR |  | TTCTAGCTCTAAAACTGAGCATGTCGATGACGG |
| *sodA*-sgF | *sodA* | TAATACGACTCACTATAGTAAATCATTGGATGCGG |
| *sodA*-sgR |  | TTCTAGCTCTAAAACCTACCGCATCCAATGATTT |
| *gedE*-sgF | *gedE* | TAATACGACTCACTATAGTTCACCCGTCATGTCTAG |
| *gedE*-sgR |  | TTCTAGCTCTAAAACGCCTAGACATGACGGGTG |
| *atf1*-sgF | *atf1* | TAATACGACTCACTATAGACGGTGAGTTCTCTTG |
| *atf1*-sgR |  | TTCTAGCTCTAAAACGAATCAAGAGAACTCACCGT |
| *ccp1*-sgF | *ccp1* | TAATACGACTCACTATAGGTCTTCTAACACCCTCC |
| *ccp1*-sgR |  | TTCTAGCTCTAAAACACAGGAGGGTGTTAGAAG |
| *napA*-sgF | *napA* | TAATACGACTCACTATAGTTACCTTTCGCCTGACC |
| *napA*-sgR |  | TTCTAGCTCTAAAACGCTGGTCAGGCGAAAGG |
| *prxA.*P-sgF | *prxA.*P | TAATACGACTCACTATAGGTAAGATAGTGGTTGTTGG |
| *prxA.*P-sgF |  | TTCTAGCTCTAAAACACCAACAACCACTATCTTAC |
| *gtr1.*P-sgF | *gtr1.*P | TAATACGACTCACTATAGGATTATTGACGGGCAC |
| *gtr1.*P-sgR |  | TTCTAGCTCTAAAACTCGGGTGCCCGTCAATAATC |
| *trxR.*P-sgF | *trxR.*P | TAATACGACTCACTATAGGCGTCGAGAACGAGTTTG |
| *trxR.*P-sgR |  | TTCTAGCTCTAAAACATCAAACTCGTTCTCGACGC |
| *grx5.*P-sgF | *grx5.*P | TAATACGACTCACTATAGATCTTACCAAACCAGCC |
| *grx5.*P-sgR |  | TTCTAGCTCTAAAACCAAGGCTGGTTTGGTAAG |
| *ccp1*.P-sgF | *ccp1.*P | TAATACGACTCACTATAGGAGGAGGAGAAGAGG |
| *ccp1*.P-sgR |  | TTCTAGCTCTAAAACCTTATCCTCTTCTCCTC |
| *catB*.P-sgF | *catB.*P | TAATACGACTCACTATAGTACCTCACCATGCGAGC |
| *catB*.P-sgR |  | TTCTAGCTCTAAAACAGAGCTCGCATGGTGAGG |
| *ccp1*.T-sgF | *ccp1.*T | TAATACGACTCACTATAGTTTGTTAGTGTGTTCAGC |
| *ccp1*.T-sgR |  | TTCTAGCTCTAAAACCAGCTGAACACACTAAC |
| *prxA*.T-sgF | *prxA.*T | TAATACGACTCACTATAGAGGTAATGACCTGTGAAG |
| *prxA*.T-sgR |  | TTCTAGCTCTAAAACGTCTTCACAGGTCATTACCT |

**Table S6 Primers used for strains construction**

| **Primer** | **Template** | **Nucleotide sequence (5’ to 3’)** |
| --- | --- | --- |
| **Gene Disruption** | | |
| pyrG.F | pUC19-*pyrG* | GAATTCGATACCTGTCG |
| pyrG.R |  | TCAGTGCTTGTCTACCAG |
| pyroA.F | pUC19-*pyroA* | CTGCAGAAGTGCGCGAAAGC |
| pyroA.R |  | GGATCCAGGAGTATACGGG |
| catA-up.F | FGSC A4 | GAGCTTTTCTTTGACCTGTCG |
| catA-up.R |  | CTGGTAGACAAGCACTGAGAGGATCGATCAGATTG |
| catA-down.F | FGSC A4 | CGACAGGTATCGAATTCTCTCACTGCCTCGGTACTTG |
| catA-down.R |  | GAAATTCTGGTGACGAATGGG |
| fu-catA.F |  | GTCGGGGAGGTTCTTCTC |
| fu-catA.R |  | GTAGTCGAAGTGGTTAAGG |
| catB-up.F | FGSC A4 | GGCATGCTCGATGTCGATC |
| catB-up.R |  | CTTTCGCGCACTTCTGCAGCACCTGGCAGGCACGGGAG |
| catB-down.F | FGSC A4 | ACCCGTATACTCCTGGATCCGAGACTAGTAGCCGAGAG |
| catB-down.R |  | CTGCCATATGACTGCTAG |
| fu-catB.F |  | GTTGCTATTCTGGGATTG |
| fu-catB.R |  | CTCTTCCATTCACTCCGTC |
| catC-up.F | FGSC A4 | GAGGGCCGAGAACATAC |
| catC-up.R |  | CGACAGGTATCGAATTCTGTGATGGTGTTGAAGCAATG |
| catC-down.F | FGSC A4 | CTGGTAGACAAGCACTGACTTAGTCACACTACTTTGG |
| catC-down.R |  | CGTTTACGATACCGTTGC |
| fu-catC.F |  | GTGACTGCCTAGCATACG |
| fu-catC.R |  | GATGACTTGCCGCAATGTG |
| napA-up.F | FGSC A4 | GCCCACTGATTTTCCCGCTC |
| napA-up.R |  | CTTTCGCGCACTTCTGCAGTATGGATATGCTATAATGTAC |
| napA-down.F | FGSC A4 | CCCGTATACTCCTGGATCCCGGATACCCACGATATGATAC |
| napA-down.R |  | CGGACGTCCTTAATTGAATG |
| fu-napA.F |  | CTGAATTTGCGGCGTAAAC |
| fu-napA.R |  | GCTTGTCCGCCTTTTTCCAG |
| ccp1-up.F | FGSC A4 | CTAGCCTGACAGTGGCATAG |
| ccp1-up.R |  | CTGGTAGACAAGCACTGAGTGGACAAGACAAGGGCAG |
| ccp1-down.F | FGSC A4 | CGACAGGTATCGAATTCGCTTCTACTCAGCGACAG |
| ccp1-down.R |  | CCAACAACAATCTCGCCC |
| fu-ccp1.F |  | CATTGTTGAACAGCAGGAC |
| fu-ccp1.R |  | CCCTCATCACAATCCAGATC |
| sodA-up.F | FGSC A4 | CTCTCTAGCTGTGAATGC |
| sodA-up.R |  | CTTTCGCGCACTTCTGCAGGAGATCGCCACTGTGAGATC |
| sodA-down.F | FGSC A4 | CCCGTATACTCCTGGATCCGATAATGTGGGCGACGAG |
| sodA-down.R |  | CTCCTGAACGACGATCTC |
| fu-sodA.F |  | GCTATGGTGGGGTTACTC |
| fu-sodA.R |  | GACAACCTTGCCGACAAG |
| gedE-up.F | FGSC A4 | CAGTCTCCTGAGCACTTG |
| gedE-up.R |  | CTTTCGCGCACTTCTGCAGCCCGCTCATTATCTACAC |
| gedE-down.F | FGSC A4 | ACCCGTATACTCCTGGATCCTTATGGACTTTCCTGGAC |
| gedE-down.R |  | CTGAAGAGCAGCGATGTC |
| fu-gedE.F |  | GTTCCACCGTGCAGATGTTC |
| fu-gedE.R |  | GACTGGCACATAGACCTTC |
| atf1-up.F | FGSC A4 | GTGCGCGATCAAGTTGTGTG |
| atf1-up.R |  | CGACAGGTATCGAATTCATAAATCAGCTGAATAATAG |
| atf1-down.F | FGSC A4 | CTGGTAGACAAGCACTGAGCAACGGTTTCTGTATTTG |
| atf1-down.R |  | CGGTCCTGACGAAAATAC |
| fu-atf1.F |  | GTCGATTCATTGTCGAG |
| fu-atf1.R |  | CTTGTACATATGGCAG |
| **Tagged Expression** | | |
| FLAG-trpC.F | pUC19-FLAG-T*trpC*-*pyroA* | GACTATAAGGACGATGACGATAAGTAATAGTGATTTAATAGCTCC |
| pyroA-TprxA.R |  | CTGCAGAAGTGCGCGAAAG |
| prxA.F | FGSC A4 | CTTAAGGCCGGTGACAGC |
| prxA-FLAG.R |  | TTACTTATCGTCATCGTCCTTATAGTCAGAGCCTCCACCCCCCAGGTGCTTGATGACAGTC |
| TprxA.F | FGSC A4 | CTTTCGCGCACTTCTGCAGCGATGCATTTACGAAGC |
| prxA-down.R |  | GTATTGTCCTCGATCATC |
| fu-prxAFLAG.F |  | CAGCTACATTCCCTGGAC |
| fu-prxAFLAG.R |  | CCAAGCGCATACCTCTCTC |
| pyroA.F | pUC19-*gfp*-T*trpC*-*pyroA* | CTGCAGAAGTGCGCGAAAGC |
| ccp1-gfp.R |  | GATATGTCTTCAAGAGGTCTGAGGGAGCTGGTGCAGGCGCTG |
| ccp1-orf.F | FGSC A4 | ATGGCTTCCGCTGCTCGATC |
| ccp1-linker.R |  | CTCAGACCTCTTGAAGACATATC |
| pyroA-Tccp1.F | FGSC A4 | GCTTTCGCGCACTTCTGCAGCGCATACCCCCTTTTCTG |
| ccp1-down.R |  | CCAACAACAATCTCGCCC |
| fu-ccp1-gfp.F |  | GATACTCTTCCGAGGCG |
| fu-ccp1-gfp.R |  | CCCTCATCACAATCCAG |
| **Primer** | **Template** | **Nucleotide sequence (5’ to 3’)** |
| **ccp1 Overexpression** | | |
| ccp1-up.F | FGSC A4 | CTAGCCTGACAGTGGCATAG |
| Pccp1-pyroA.R |  | CTTTCGCGCACTTCTGCAGCAGGAAGGACAGGGATAC |
| pyroA.F | pUC19-*pyroA* | CTGCAGAAGTGCGCGAAAGC |
| pyroA-PgpdA.R |  | CTCCGACTTCAATAAGGAGCGGATCCAGGAGTATACGGG |
| PgpdA.F | pUC19-*pyroA*-P*gpdA*-*ccp1*-T*trpC* | GCTCCTTATTGAAGTCGGAG |
| ccp1-orf.R |  | TTACTCAGACCTCTTGAAGAC |
| fu-Pccp1.F |  | CATTGTTGAACAGCAGGAC |
| fu-Pccp1.R |  | CTTGTCCTGGACAAGTG |
| **MTS-catB expression** | | |
| catB-up.F | FGSC A4 | GCATGCTCGATGTCGATC |
| PcatB-pyroA.R |  | CTTTCGCGCACTTCTGCAGGAAAGAGGGATAGGAATG |
| pyroA.F | pUC19-*pyroA* | CTGCAGAAGTGCGCGAAAGC |
| pyroA-PgpdA.R |  | CTCCGACTTCAATAAGGAGCGGATCCAGGAGTATACGGG |
| PgpdA.F | pUC19-*pyroA*-P*gpdA*-*ccp1*-T*trpC* | GCTCCTTATTGAAGTCGGAG |
| ccp1-MTS.R |  | GTATCCACGGCGAGAAGCAAC |
| MTS-McatB.F | FGSC A4 | GTTGCTTCTCGCCGTGGATACGTCTGTCCGTATATGACAG |
| catB.R |  | CATACCTCCCACTCAG |
| fu-McatB.F |  | GAGAACTGCCGATCTCTC |
| fu-McatB.R |  | GACCATACTTGCCAAACC |
| **Low-expression strain** | | |
| PniaD.F | FGSC A4 | ATGGCGGGCGCGGTGATTGAGC |
| PniaD.R |  | TGTGAGAGTATGGGATAGG |
| prxA-up.F | FGSC A4 | GCGTATATCGAGGCAACC |
| prxA-pyrG.R |  | CGACAGGTATCGAATTCGTGTAGAGAAGTAGGAG |
| pyrG.F | pUC19-*pyrG* | GAATTCGATACCTGTCG |
| pyrG-PniaD.R |  | CTGGTAGACAAGCACTGAATGGCGGGCGCGGTGATTGAGC |
| PniaD-prxA.F | FGSC A4 | CCTATCCCATACTCTCACAATGTCTGGACTTAAGGCC |
| prxA.R |  | GCTGGAGAACTCAAGGTG |
| fu-PprxA.F |  | GGCGGTCGATCATTTCTC |
| fu-PprxA.R |  | CCGATGCTCTTGGAGAAC |
| trxR-up.F | FGSC A4 | TATTGTGGGTAGGTTATTC |
| trxR-pyroA.R |  | CTTTCGCGCACTTCTGCAGGACGATGTGGGAGAGTTC |
| pyroA.F | pUC19-*pyroA* | CTGCAGAAGTGCGCGAAAGC |
| pyroA-PniaD.R |  | GCTCAATCACCGCGCCCGCCATGACGATGTGGGAGAGTTC |
| PniaD-trxR.F | FGSC A4 | CTATCCCATACTCTCACAATGGTTCACTCCAAAG |
| trxR.R |  | CTTGGTAGCGATGTATCCGTCCTC |
| fu-trxR.F |  | CGATTCCTCGGTGTTGAG |
| fu-trxR.R |  | GTTAGCCTCTAGGGTC |
| gtr1-up.F | FGSC A4 | CTTCGTCAGGGCGTGTCTTG |
| Pgtr1-pyrG.R |  | AGAAGCACTTACCTTCGCATGCGAGCTTAGGCGCAGGATG |
| PniaD-gtr1.F | FGSC A4 | CCTATCCCATACTCTCACAATGCTCTCTCGCTCCTCGCTTCG |
| gtr1.R |  | TTGTCCTTGCCCTTGCCGTC |
| fu-gtr1.F |  | GGATAACCCTGACGTAC |
| fu-gtr1.R |  | GTCATGGTCTTCTGGATC |
| grx5-up.F | FGSC A4 | CGTGTAATCCTCCACTGC |
| grx5-pyrG.R |  | CTGTCCATAGCTTAAGTACACGGCAGCAATCAC |
| PniaD-grx5.F | FGSC A4 | CCTATCCCATACTCTCACAATGTTCTCAAGAACAGC |
| grx5.R |  | CCCACTCACCTATGATC |
| fu-grx5.F |  | GAAGAAGGTAGACCCAGC |
| fu-grx5.R |  | GATCCTTAAGAGCAGAGAG |

**Table S7 Primers used for strains verification**

| **Primers** | **Gene location** | **Nucleotide sequence (5’ to 3’)** |
| --- | --- | --- |
| pyrG-check.F | *pyrG* | CTCACTGACGGCTCTAGTAG |
| pyrG-check.R | *pyrG* | TAGGAGAGACCCGTGTTCCG |
| pyroA-check.F | *pyroA* | GCTGGTGAGAACACATGC |
| pyroA-check.R | *pyroA* | CGCAGGGACTATATTGC |
| catA-up.F | *catA*.P | GAGCTTTTCTTTGACCTGTCG |
| catA-down.R | *catA*.T | GAAATTCTGGTGACGAATGGG |
| catA-orf.F | *catA* | CTACTAGTATCACCGCCG |
| catA-orf.R | *catA* | CAACCGTCGAAGCCAATC |
| catB-up.F | *catB*.P | GGCATGCTCGATGTCGATC |
| catB-down.R | *catB*.T | CTGCCATATGACTGCTAG |
| catB-orf.F | *catB* | CTGGTCGGCCTTGTTG |
| catB-orf.R | *catB* | CTTGAACACGGTCAGAC |
| catB.R | *catB* | CATACCTCCCACTCAG |
| catC-up.F | *catC*.P | GAGGGCCGAGAACATAC |
| catC-down.R | *catC*.T | CGTTTACGATACCGTTGC |
| catC-orf.F | *catC* | ATGGGCCAAAACGACGAC |
| catC-orf.R | *catC* | CTACAAACGAGCCCTGG |
| napA-up.F | *napA*.P | GCCCACTGATTTTCCCGCTC |
| napA-down.R | *napA*.T | CGGACGTCCTTAATTGAATG |
| napA-orf.F | *napA* | ATGGCCGACTACAATTC |
| napA-orf.R | *napA* | CTACACGCGGCCAATG |
| ccp1-up.F | *ccp1*.P | CTAGCCTGACAGTGGCATAG |
| ccp1-down.R | *ccp1*.T | CCAACAACAATCTCGCCC |
| ccp1-orf.F | *ccp1* | ATGGCTTCCGCTGCTCGATC |
| ccp1-orf.R | *ccp1* | TTACTCAGACCTCTTGAAGAC |
| sodA-up.F | *sodA*.P | CTCTCTAGCTGTGAATGC |
| sodA-down.R | *sodA*.T | CTCCTGAACGACGATCTC |
| sodA-orf.F | *sodA* | GTTCTTAGTTGCTGTCC |
| sodA-orf.R | *sodA* | GGGTGAATTTACGCAGC |
| gedE-up.F | *gedE*.P | CAGTCTCCTGAGCACTTG |
| gedE-down.R | *gedE*.T | CTGAAGAGCAGCGATGTC |
| gedE-orf.F | *gedE* | GTAGATAATGAGCGGGG |
| gedE-orf.R | *gedE* | CGAGCTCAACTGACTG |
| atf1-up.F | *atf1*.P | GTGCGCGATCAAGTTGTGTG |
| atf1-down.R | *atf1*.T | CGGTCCTGACGAAAATAC |
| atf1-orf.F | *atf1* | CTTCGGCAGTCTCAAC |
| atf1-orf.R | *atf1* | GTGTATGGAGGATTCGG |
| prxA.F | *prxA* | CTTAAGGCCGGTGACAGC |
| prxA-down.R | *prxA*.T | GTATTGTCCTCGATCATC |
| prxA-up.F | *prxA*.P | GCGTATATCGAGGCAACC |
| prxA.R | *prxA* | GCTGGAGAACTCAAGGTG |
| trxR-up.F | *trxR*.P | TATTGTGGGTAGGTTATTC |
| trxR.R | *trxR* | CTTGGTAGCGATGTATCCGTCCTC |
| gtr1-up.F | *gtr1*.P | CTTCGTCAGGGCGTGTCTTG |
| gtr1.R | *gtr1* | TTGTCCTTGCCCTTGCCGTC |
| grx5-up.F | *grx5*.P | CGTGTAATCCTCCACTGC |
| grx5.R | *grx5*.T | CCCACTCACCTATGATC |
| PgpdA.F | *gpdA*.P | GCTCCTTATTGAAGTCGGAG |
| Hyper7.R | *HyPer7* | TTAGTCGCAGATGAAGG |

**Table S8 Primers used for qRT-PCR**

| **Primers** | **Gene** | **Nucleotide sequence (5’ to 3’)** |
| --- | --- | --- |
| Qactin.F | *actin* | GAAGTCCTACGAACTGCCTGATG |
| Qactin-R |  | GACCAAGAACGCTGGGCTGG |
| QprxA.F | *prxA* | CCCCGCTGACGTTGTCTTC |
| QprxA.R |  | GAGGGCGAAGAGGATGACC |
| QtrxR.F | *trxR* | GTTATCATCGGCTCCGGC |
| QtrxR.R |  | CGACATCAGTGGTCCGTGG |
| Qccp1.F | *ccp1* | CTATGGACCTGTCCTTGTCC |
| Qccp1.R |  | GGAATTTGGCCTTGATGGGC |
| QcatB.F | *catB* | GACCAGCAGAGTCTCAAG |
| QcatB.R |  | GGTGATGTTGGAGAAGTC |
| QtrxA.F | *trxA* | ATGGGTGCCTCTGAACACG |
| QtrxA.R |  | ATGTCGCGAAGCAGTCGACG |
| QsodA.F | *sodA* | GTCGGTGACCTTGGTAAC |
| QsodA.R |  | CAAGGGTCCGGCCCAGAAC |
| QgedE.F | *gedE* | CAGAAGGAGCCCTGGTTC |
| QgedE.R |  | GGTAAGGATACTACCGGAC |
| Qgtr1.F | *gtr1* | CGTTAATGTTGGCTGCGTC |
| Qgtr1.R |  | GTATACGCCATTCAACCGC |
| Qgrx5.F | *grx5* | CGTCAAGGTATCAAGGAG |
| Qgrx5.R |  | CTGATGCATTGACATCAG |
| QcatC.F | *catC* | CGACCAAAAGACGTACCGC |
| QcatC.R |  | GCTCGGGAATGCGCTCAC |
| Qgpx2.F | *gpx2* | CCTCTTCGACCAGACAAGTC |
| Qgpx2.R |  | GTTCGATGTGAGGGGAAGTG |
| QcatA.F | *catA* | CTGACGAGGGAAATTGGG |
| QcatA.R |  | GTGCAAGTACACAAAGTC |
| QtpsA.F | *tpsA* | GGGTTTTCTAACTCCATCC |
| QtpsA.R |  | CTGAACTTGGCTCGCAACAG |
| Qatf1.F | *atf1* | GATGGATAACGAGGCCTCATC |
| Qatf1.R |  | CTGAGTTGAAGTAATCGGGG |
| QnapA.F | *napA* | GAGGGAGAGTGATGACAAG |
| QnapA.R |  | CACTTTCGCTTCCAGATCC |

**Supplementary References**

1. Li, J., Sun, Y., Liu, F., Zhou, Y., Yan, Y., Zhou, Z., Wang, P., and Zhou, S. (2022). Increasing NADPH impairs fungal H(2)O(2) resistance by perturbing transcriptional regulation of peroxiredoxin. Bioresour Bioprocess *9*, 1. 10.1186/s40643-021-00489-w.

2. Zhou, Y., Lv, H., Li, H., Li, J., Yan, Y., Liu, F., Hao, W., Zhou, Z., Wang, P., and Zhou, S. (2021). Nitroreductase Increases Menadione-Mediated Oxidative Stress in Aspergillus nidulans. Appl Environ Microbiol *87*, e0175821. 10.1128/aem.01758-21.

3. Yan, Y., Huang, X., Zhou, Y., Li, J., Liu, F., Li, X., Hu, X., Wang, J., Guo, L., Liu, R., et al. (2023). Cytosol Peroxiredoxin and Cell Surface Catalase Differentially Respond to H(2)O(2) Stress in Aspergillus nidulans. Antioxidants (Basel) *12*. 10.3390/antiox12071333.

4. Liu, F., Guo, L., Luo, Y., Li, J., Zhou, Y., Wang, J., Huang, X., Tan, X., Fu, M., Yu, B., et al. (2025). Peroxiredoxin PrxA and thioredoxin TrxA mediate the redox signal to the transcription factor NapA in the fungus Aspergillus nidulans. Int J Biol Macromol *310*, 143434. 10.1016/j.ijbiomac.2025.143434.
