# Supplementary material for: Fungal oxidative stress tolerance depends on peroxiredoxin PrxA-mediated redox signaling to mitochondrial cytochrome c peroxidase Ccp1
Source: J Biol Chem. 2026 Apr 27;302(6):113084. doi: 10.1016/j.jbc.2026.113084 (PMC13253075; doi:10.1016/j.jbc.2026.113084)
Supplement: Supporting information file [file mmc3.docx]

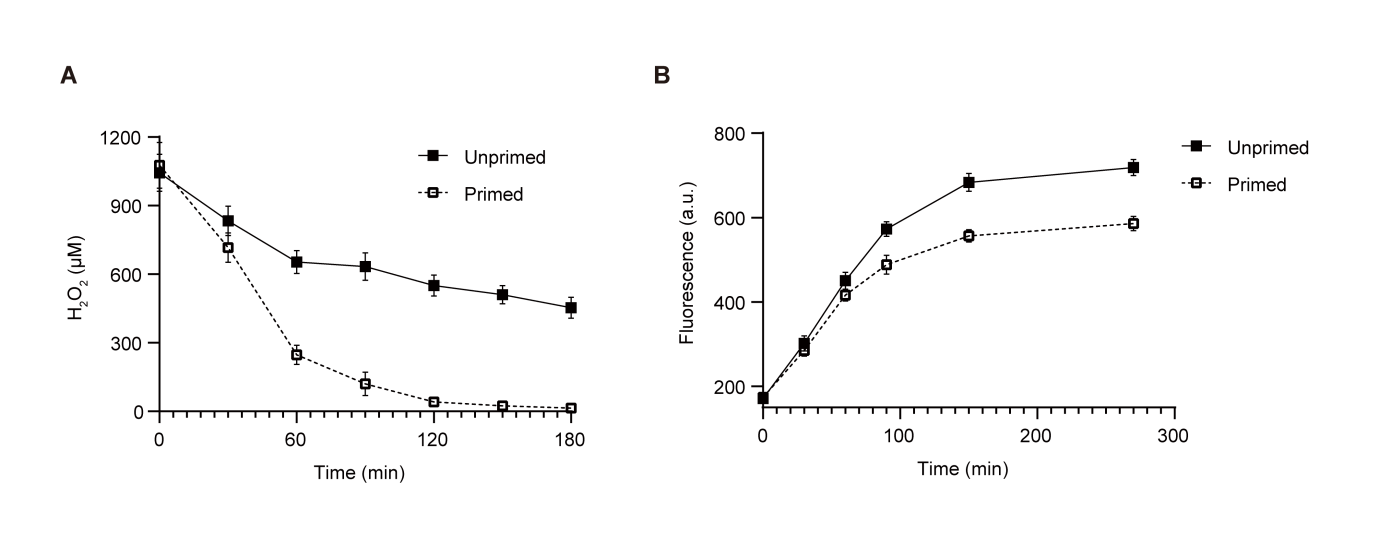


**Figure S1. Quantification of extracellular and intracellular H_2_O_2_.**

**(A)** Extracellular H_2_O_2_ concentration curve. Wild-type strains were inoculated into MM medium containing nitrate as the sole nitrogen source and cultured at 37 °C for 4.5 h to obtain short germlings. The priming group was treated with 0.5 mM H_2_O_2_ for 30 min, after which 1 mM H_2_O_2_ was added to both the primed and non-primed groups for oxidative challenge. At 30 min intervals, 1 mL of culture supernatant was collected and measured using a commercial H_2_O_2_ assay kit (Beyotime S0038), and H_2_O concentrations were calculated from a standard curve. The absorbance at 560 nm (A_560_) was measured, and extracellular H_2_O_2_ concentrations were calculated using a standard curve. Data represent mean ± SD, n = 3. **(B)** Intracellular H_2_O_2_ quantification. Short germlings cultured for 4.5 h were incubated with the H_2_O_2_ -specific fluorescent probe BES-H_2_O_2_ -Ac for 2 h to allow probe uptake. The priming group was then treated with 0.5 mM H_2_O_2_ for 30 min, followed by the addition of 1 mM H_2_O_2_ to all groups. Samples were collected every 30 min, and fluorescence intensity was measured at 485/515 nm (Ex/Em) to quantify intracellular H_2_O_2_ levels. Data represent mean ± SD, n = 3.


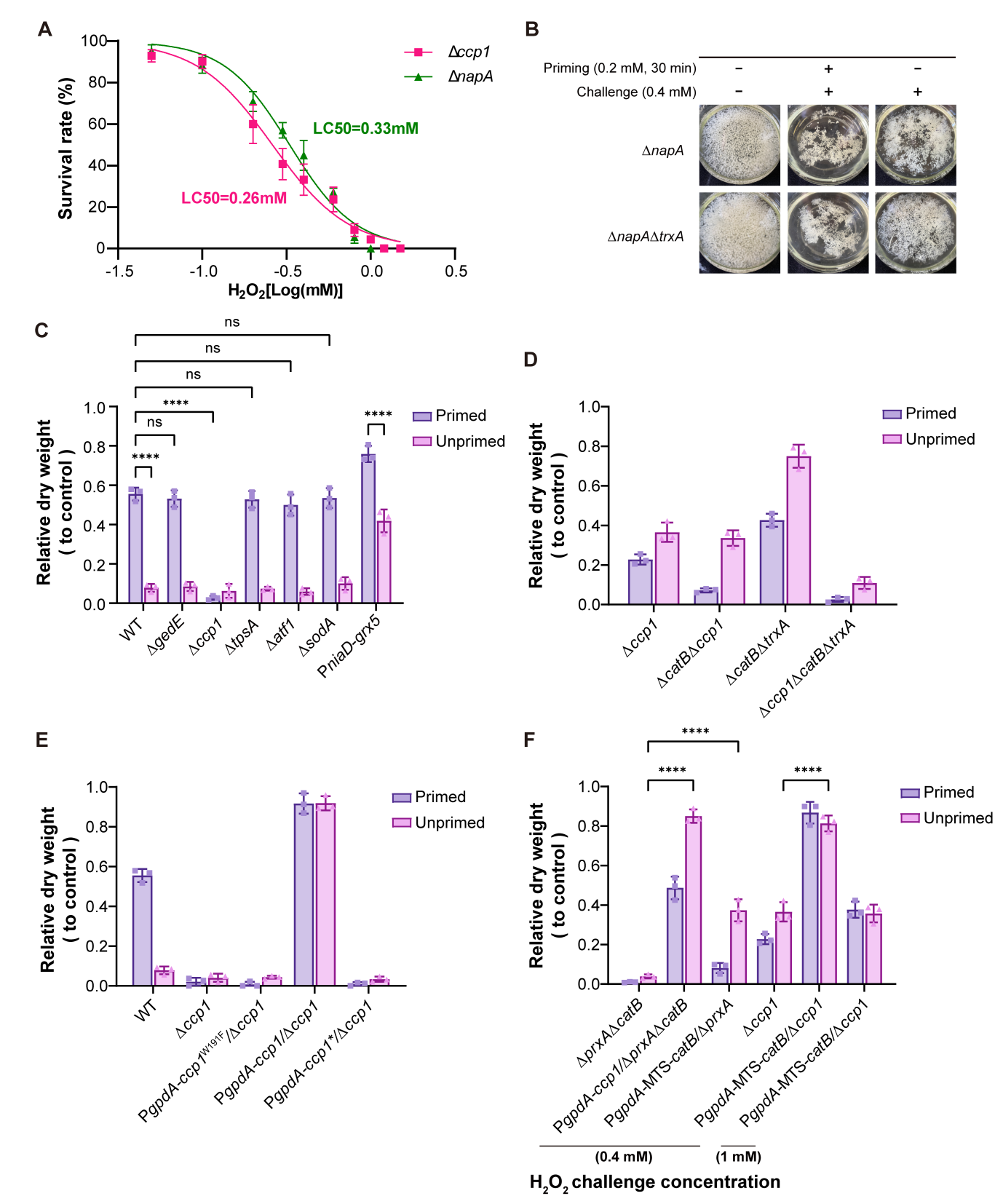


**Figure S2. Dose–response analysis and quantitative analysis of adaptive oxidative tolerance using mycelial dry weight measurements.**

(**A**) Dose–response curves and LC50 determination of Δ*napA* and Δ*ccp1* strains under increasing concentrations of H_2_O_2_. Conidia were cultured in liquid minimal medium for 4.5 h before the addition of H_2_O_2_ and further incubation for 12 h. Survival rates were calculated based on mycelial dry weight. LC50 values were determined by nonlinear regression analysis. Data represent mean ± SD from three independent biological replicates. (**B**) Δ*napA* and Δ*napAΔtrxA* strains were grown for 4.5 h to the short-hyphal stage at 37 °C with shaking at 220 rpm. Cultures were subjected to the same priming–challenge regimen (0.2 mM priming → 0.4 mM challenge → 12 h growth). Images are representative of three independent biological replicates. (**C**) Quantification of mycelial dry weight under priming–challenge conditions. Corresponding colony phenotypes are shown in Figure 3D. (**D**) Relative mycelial dry weight of the indicated mutants under priming–challenge treatment. Quantification corresponding to Figure 4A. (**E**) Quantification of the growth phenotypes shown in Figure 5B. (**F**) Relative mycelial dry weight under different H_2_O_2_ challenge doses. Quantification corresponding to Figure 6A. Bar graphs represent mean ± SD from three independent biological replicates (n = 3). Statistical significance was determined using two-way ANOVA with Tukey’s multiple comparison test. *P < 0.05, **P < 0.01, ***P < 0.001, ****P < 0.0001; ns, not significant.


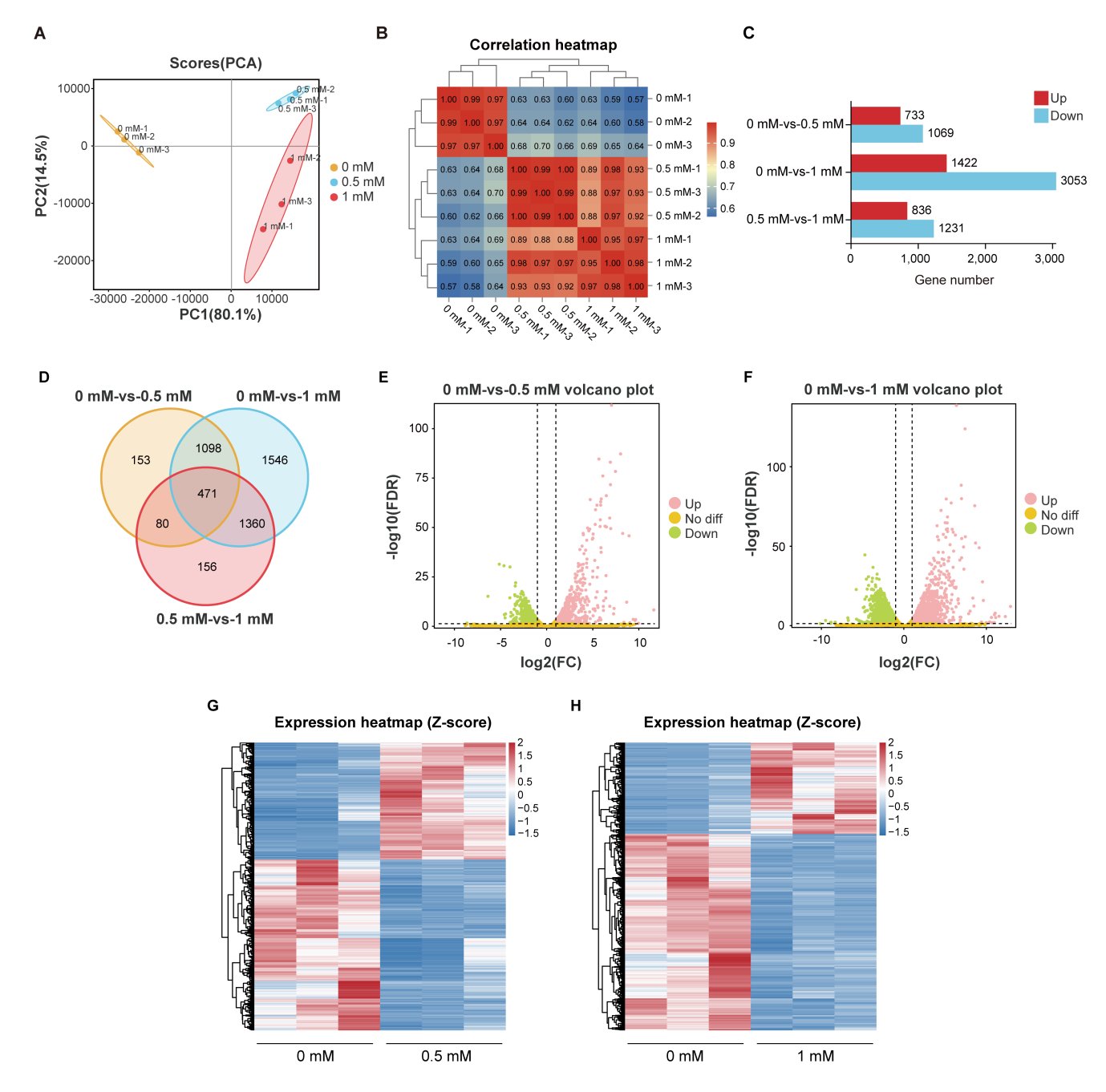


**Figure S3. PCA-based sample quality assessment and differential expression analysis of the wild-type transcriptome under varying H_2_O_2_ concentrations.**

**(A)** Principal component analysis (PCA). PCA of transcriptome profiles from Control, 0.5 mM H_2_O_2_, and 1 mM H_2_O_2_ samples. PC1 and PC2 represent the first two principal components and capture the major sources of expression variance across treatments. **(B)** Sample correlation heatmap. Pearson correlation coefficients of global expression profiles among all samples. Hierarchical clustering depicts relationships based on overall transcriptional similarity and indicates dataset-level consistency. **(C)** Quantification of differentially expressed genes (DEGs) across treatment groups. Bar plots summarize the numbers of up- and down-regulated genes identified in pairwise comparisons among Control, 0.5 mM H_2_O_2_, and 1 mM H_2_O_2_ samples. **(D)** Overlap of DEGs among comparison groups. The Venn diagram displays shared and uniquely regulated genes across the three comparisons, revealing both a core set of commonly responsive genes and subsets exhibiting concentration-specific transcriptional regulation. **(E-F)** Volcano plots of differential expression. Volcano plots illustrate the distribution of log₂ fold-changes and statistical significance (–log_10_ FDR) for the Control vs. 0.5 mM (**E**) and Control vs. 1 mM (**F**) comparisons. Up-regulated, down-regulated, and non-differential genes are displayed in pink, green, and yellow, respectively (threshold: |log_2_ FC| > 1, FDR < 0.05), providing an overview of the magnitude and directionality of transcriptional changes. **(G-H)** Heatmaps of expression patterns of significant DEGs. Heatmaps depict Z-score–standardized expression profiles of significantly regulated genes for each comparison, with hierarchical clustering illustrating coherent expression trends and regulatory patterns across treatments.

**
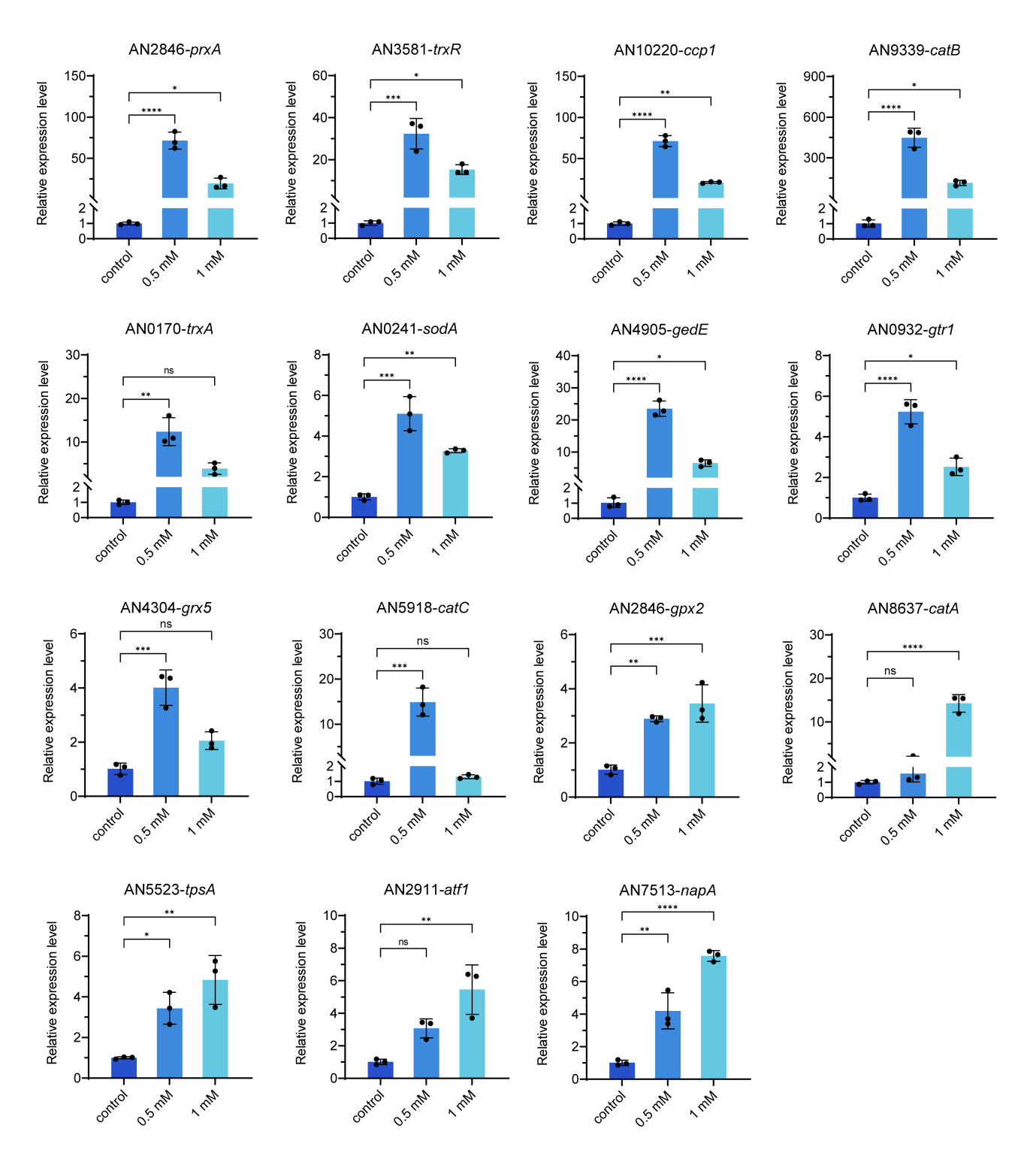
**

**Figure S4. qRT-PCR validation of transcriptomic changes.**

Wild-type cultures were sampled under control conditions, after priming with 0.5 mM H_2_O_2_ for 30 min, and after exposure to 1 mM H_2_O_2_. Transcript levels of the selected genes were quantified by qRT-PCR. Data were analysed using the 2^-ΔΔCt^ method, normalized to *actin* expression within each sample, and expression levels compared to untreated conditions (control). Bars represent mean values from three biological replicates (n = 3), with SD shown as error bars. P values were determined using one-way ANOVA followed by Dunnett’s multiple comparisons test (****P < 0.0001; ns, not significant).


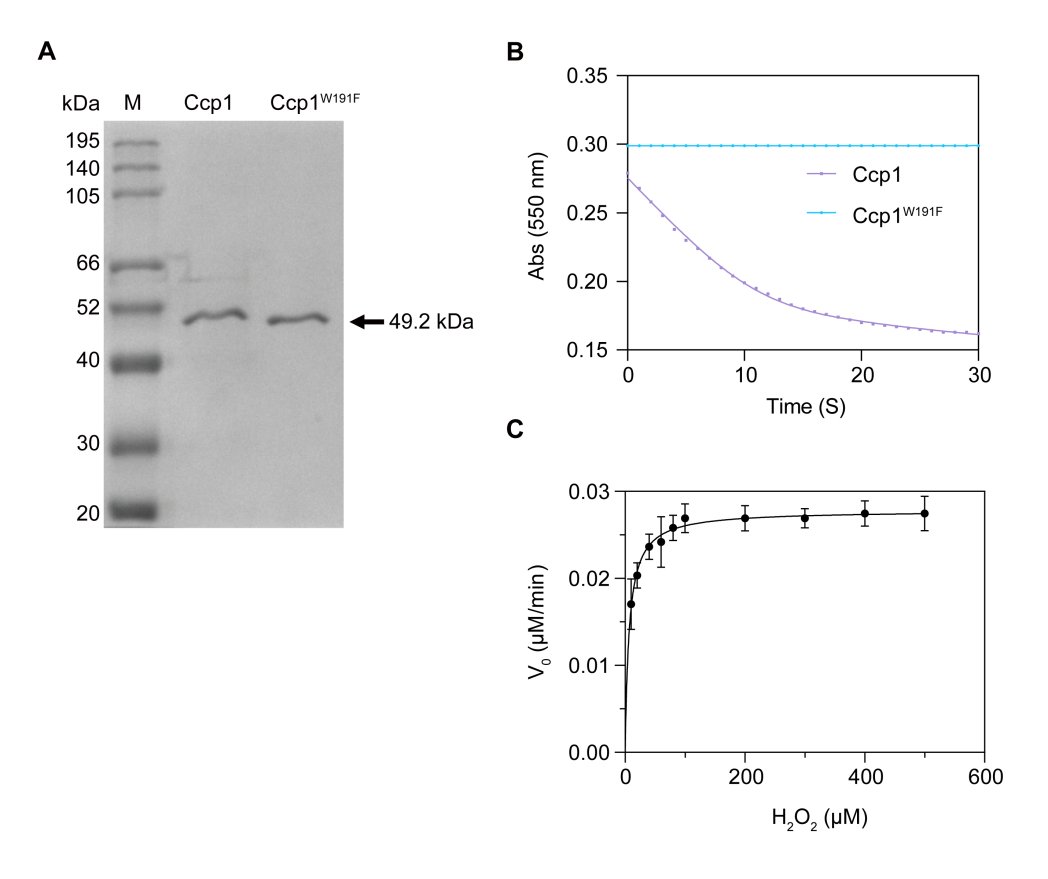


**Figure S5. Expression, purification, and enzymatic characterization of recombinant Ccp1 and its mutant Ccp1^W191F^.**

**(A)** SDS–PAGE analysis of recombinant Ccp1 and Ccp1^W191F^. The pET-28a-SUMO-*ccp1* and pET-28a-SUMO-*ccp1*^W191F^ constructs were expressed in *E. coli* BL21(DE3) at 30 ℃ with 0.2 mM IPTG induction, followed by Ni^2+^-affinity purification. Purified proteins were analyzed on a 10% SDS–PAGE gel. M, protein molecular-weight marker. **(B)** Cytochrome c peroxidase activity assay. Reactions were performed at 25 ℃ in a 1 mL mixture containing 50 mM PBS (pH 7.0), 40 µM reduced cytochrome c, 8 µg purified Ccp1 (or Ccp1^W191F^), and initiated by adding 100 µM H_2_O_2_. The reaction rate was monitored by the time-dependent decrease in absorbance of cytochrome c at 550 nm (A_550_). Data shown are representative of three independent experiments. **(C)** Catalytic kinetics of Ccp1. Using the same reaction conditions as in (**B**), initial rates (V_0_) were determined across a substrate gradient of 10–400 µM H_2_O_2_ by measuring the initial decline in A_550_. Initial rates were plotted against substrate concentrations to generate a Michaelis–Menten curve for kinetic parameter fitting. Each reaction contained 8 µg of Ccp1. Data represent mean ± SD from three independent experiments.


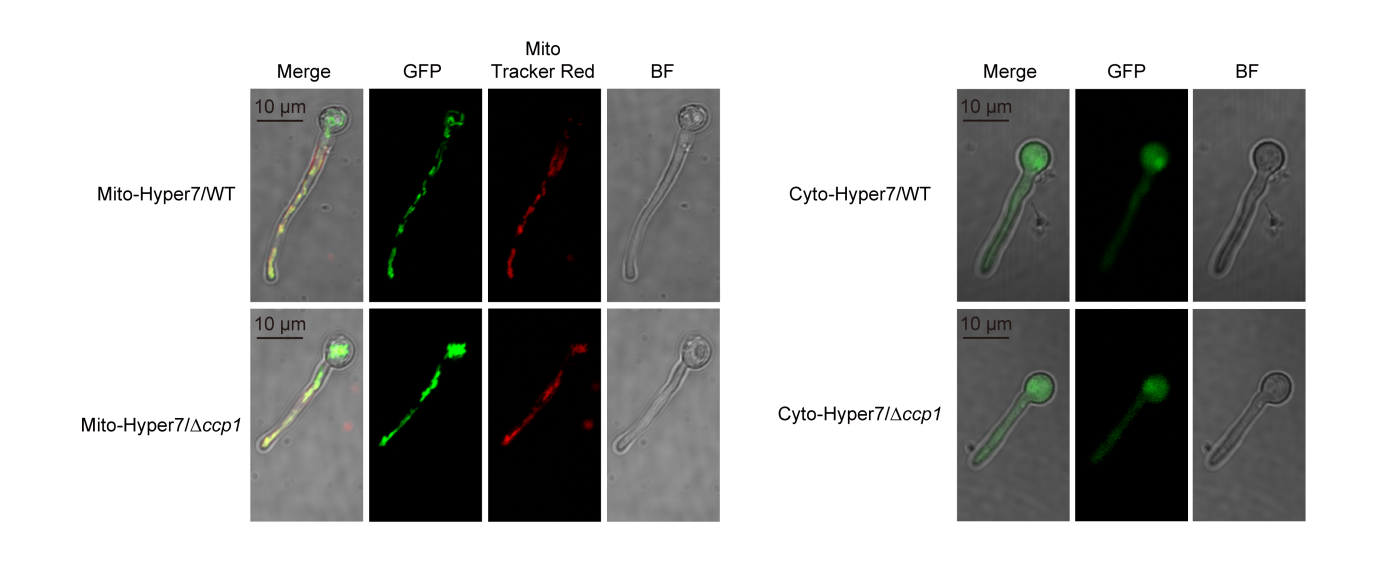


**Figure S6. Subcellular distribution of HyPer7 probes expressed in WT and Δ*ccp1* strains.**

Representative fluorescence and BF images of germlings expressing mitochondria-targeted HyPer7 (Mito-HyPer7) or cytosolic HyPer7 (Cyto-HyPer7) in the wild-type background (/WT) or the ccp1 deletion background (/Δ*ccp1*). For each strain–probe combination, BF, Mito-Tracker Red (EX=561 nm / EM=595 nm), GFP (EX=488 nm / EM=525 nm), and merged images are shown. All images were acquired under identical microscope settings. Scale bars, 10 µm.


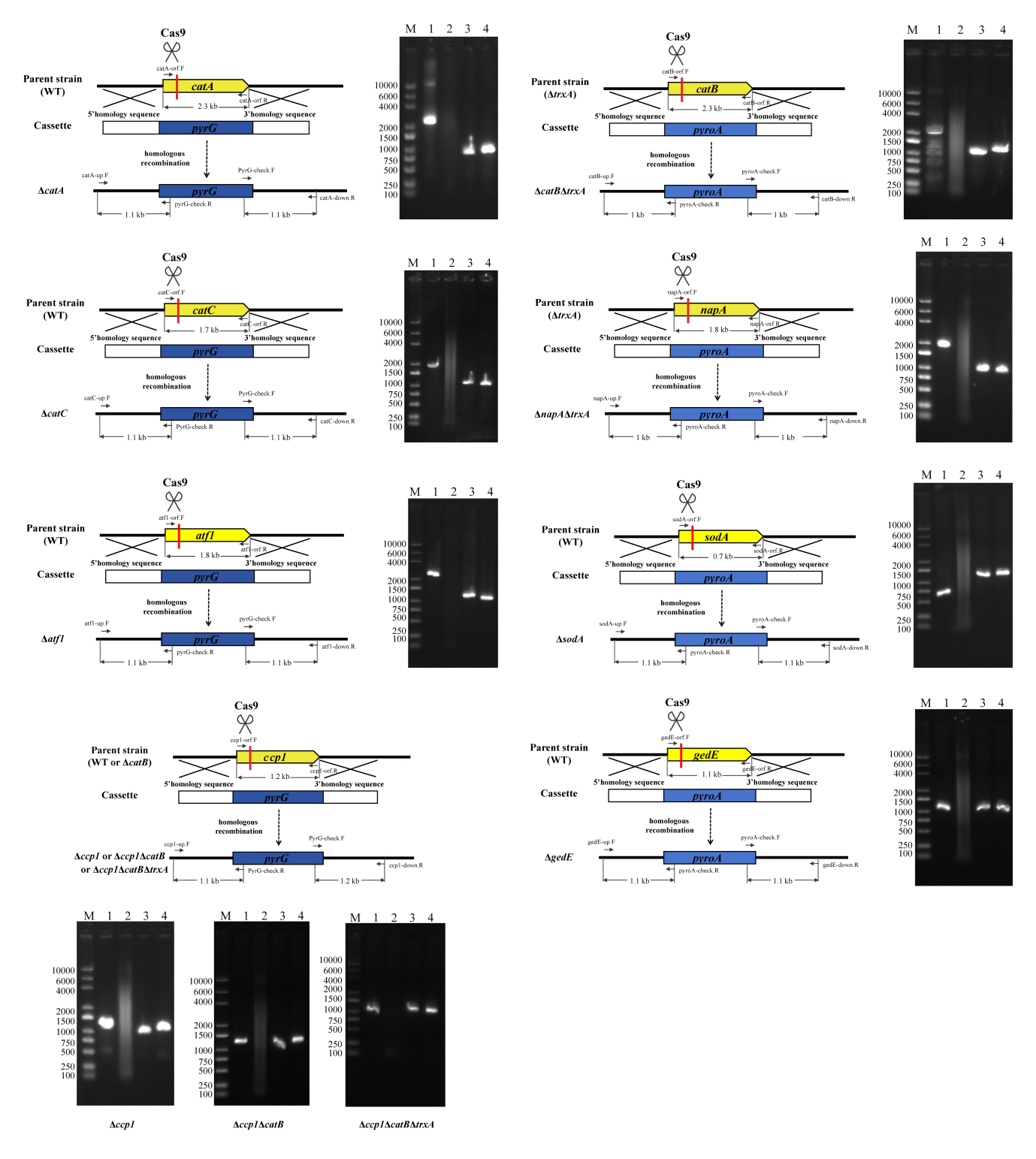


**Figure S7. Construction and verification of gene-disruption mutants in *A. nidulans*.**

The left panels illustrate the homology-directed recombination strategy used to generate the **Δ**catA, **Δ**catC, **Δ**ccp1, **Δ**ccp1**Δ**catB, **Δ**ccp1**Δ**catB**Δ**trxA, **Δ**atf1, **Δ**catB**Δ**trxA, **Δ**sodA, **Δ**gedE, and **Δ**napA**Δ**trxA mutants. Cas9-induced double-strand breaks were repaired using donor cassettes containing ***pyrG*** or ***pyroA*** flanked by the appropriate 5′ and 3′ homology arms, thereby replacing the corresponding coding regions. The right panels show PCR verification of the deletion strains. Using **Δ**catA as an example, primers **catA-up.F/catA-down.R** distinguish the parental strain (lane 1) from putative deletion isolates (lane 2), whereas **catA-up.F/pyrG-check-5′-R** (lane 3) and **pyrG-check-3′-F/catA-down.R** (lane 4) confirm correct 5′ and 3′ integration of the selection cassette. Verification of all other mutants followed the same strategy. Primer sequences are listed in Table S7.


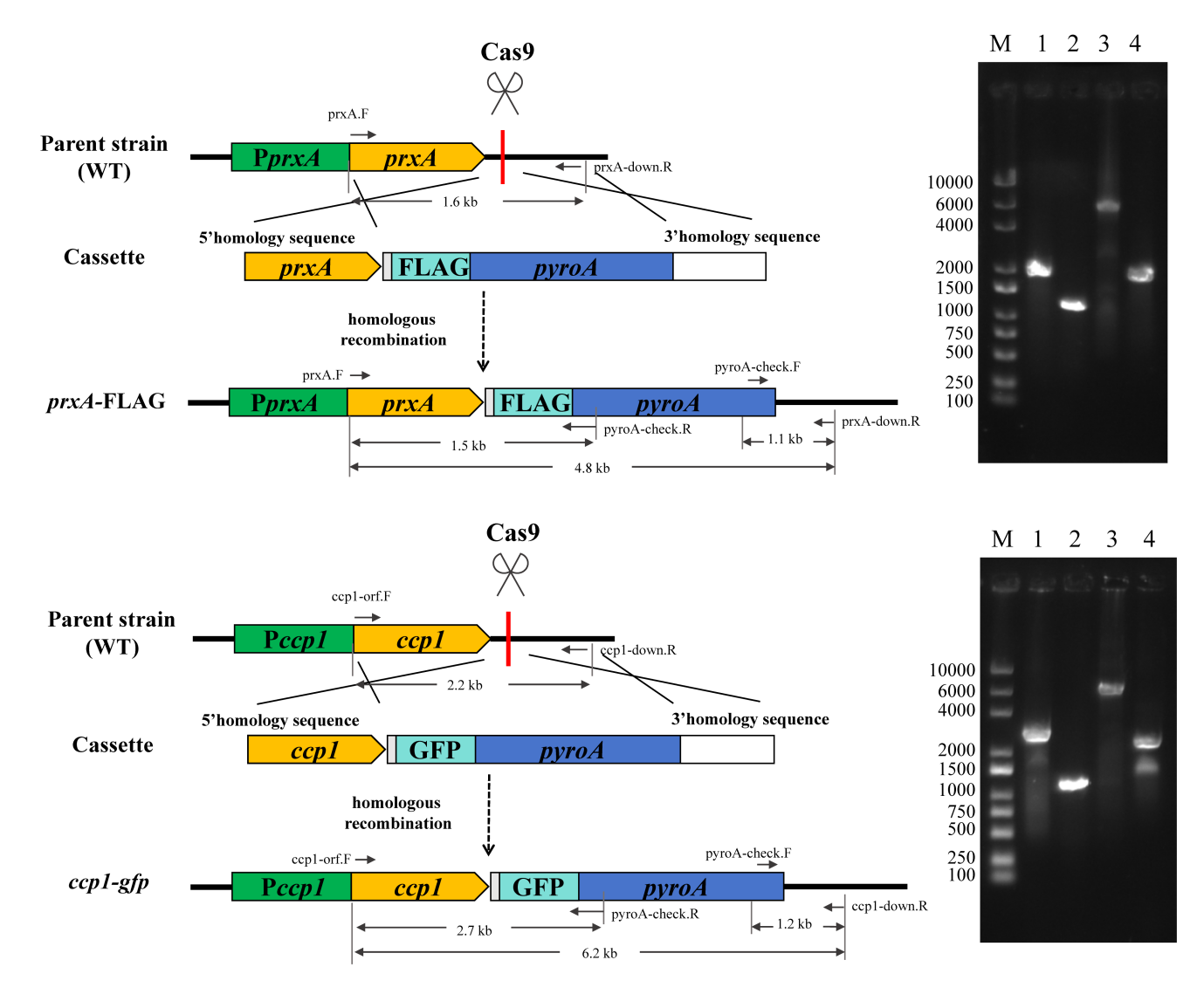


**Figure S8. Construction and validation of *prxA*-FLAG and *ccp1*-*gfp* tagged strains.**

The left panels illustrate the strategy used to generate the prxA-FLAG and ccp1-*gfp* fusion strains. CRISPR/Cas9 was employed to induce a double-strand break at the endogenous prxA or ccp1 locus, and a donor cassette containing 5′ and 3′ homology arms was introduced to direct precise C-terminal tagging through homologous recombination. The donor constructs carried an in-frame **FLAG** or **GFP** tag fused to PrxA or Ccp1, together with the ***pyroA*** selectable marker for integration screening. The right panels show PCR validation of the recombinant strains. For the prxA-FLAG strain, primer pairs **prxA.F/pyroA-check.R** (lane 1) and **pyroA-check.F/prxA-down.R** (lane 2) were used to confirm correct 5′ and 3′ side integration, respectively. The endogenous region was further amplified using **prxA.F/prxA-down.R** to distinguish the recombinant transformant (lane 3) from the parental strain(lane 4). The ccp1-gfp strain was validated using the same strategy. All primer sequences are listed in Table S7.


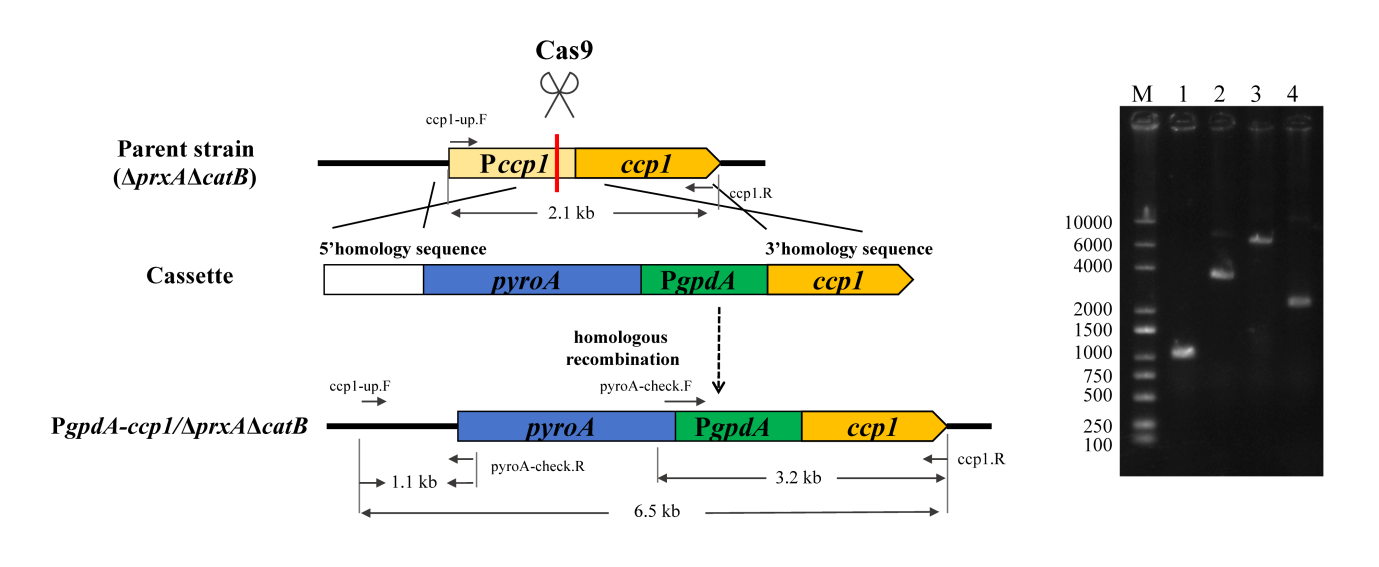


**Figure S9. Construction and PCR verification of the P*gpdA*-*ccp1* strain in the Δ*prxA*Δ*catB* background.**

The diagram illustrates replacement of the native *ccp1* promoter with the constitutive promoter P*gpdA* in the Δ*prxA*Δ*catB* background. Cas9 introduces a break upstream of *ccp1*, and a donor cassette containing *pyroA*-P*gpdA*-*ccp1* flanked by 5′ and 3′ homology arms is integrated into the genomic locus through homologous recombination. PCR verification is shown on the right: primer pairs ccp1-up.F/pyroA-check.R (lane 1) and pyroA-check.F/ccp1-R (lane 2) examine the 5′ and 3′ junctions of the integration, and primer pair ccp1-up.F/ccp1-R distinguishes the recombinant transformant (lane 3) from the parental strain (lane 4); lane M indicates the DNA size marker.


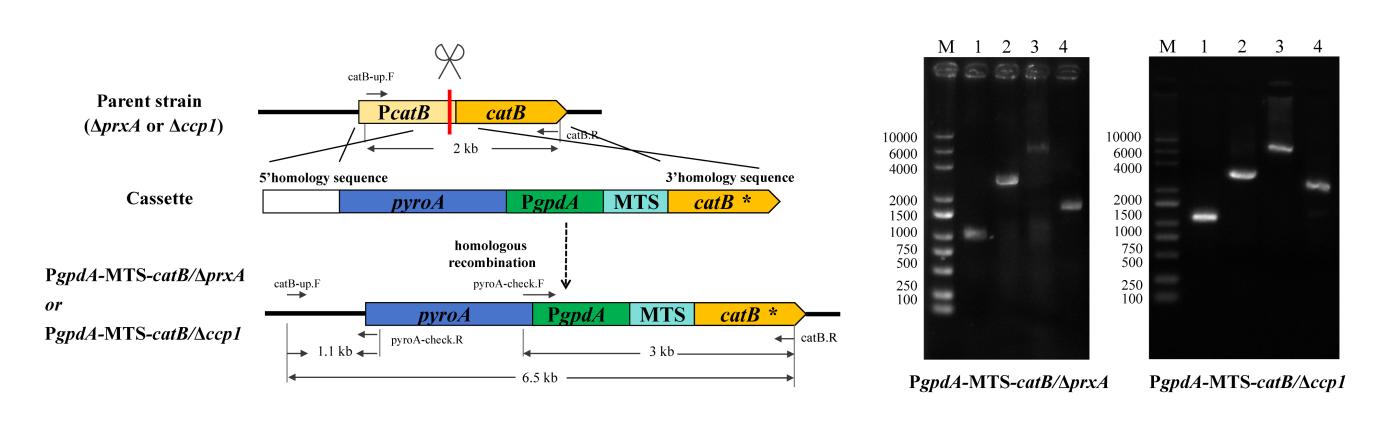


**Figure S10. Construction and PCR verification of the P*gpdA*-MTS-*catB* strain in the Δ*prxA* or Δ*ccp1* background.**

The schematic illustrates replacement of the endogenous *catB* locus with the mitochondrial-targeted construct P*gpdA*-MTS-*catB* in Δ*prxA* or Δ*ccp1* strains. Cas9 introduces a break at the native *catB* locus, and a donor cassette containing *pyroA*-P*gpdA*-MTS-*catB* flanked by 5′ and 3′ homology arms is integrated through homologous recombination. PCR verification is shown on the right: primer pairs catB-up.F/pyroA-check.R (lane 1) and pyroA-check.F/catB.R (lane 2) were used to examine the 5′ and 3′ junctions of the integration, and primer pair catB-up.F/catB.R distinguishes recombinant transformants (lane 3) from the parental strain (lane 4); lane M indicates the DNA size marker.


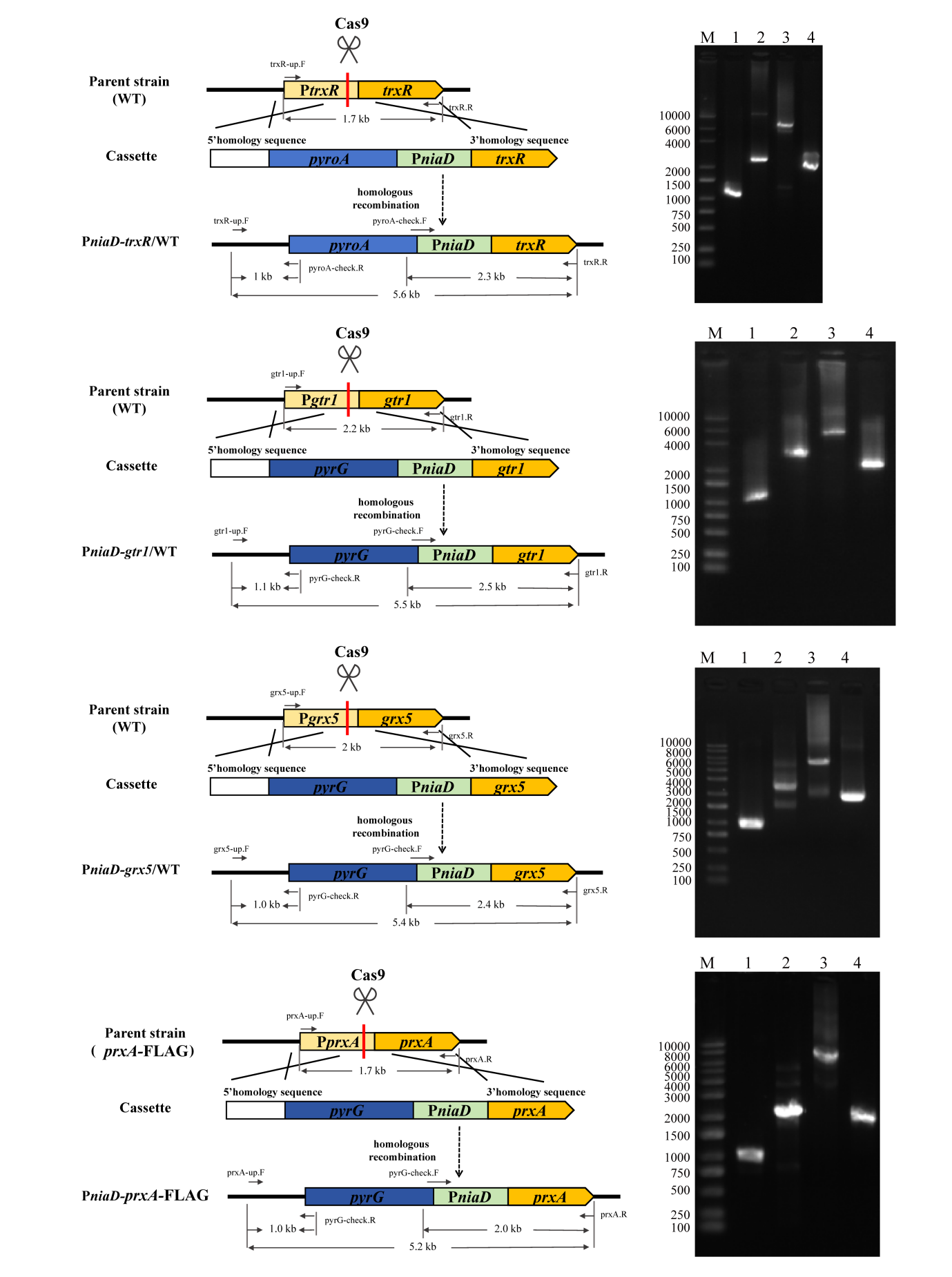


**Figure S11. Construction and PCR verification of the P*niaD-trxR*, P*niaD-gtr1*, P*niaD-grx5* strains, and P*niaD-prxA-*FLAG.**

Each schematic illustrates replacement of the native *trxR*, *gtr1*, or *grx5* locus in the WT background and *prxA* locus in the *prxA-*FLAG background by a promoter-swap cassette in which the coding region is placed under the nitrogen-repressible promoter P*niaD*. Cas9 introduces a cleavage at the endogenous locus, and donor constructs consisting of *pyroA*–P*niaD–trxR*, *pyrG*–P*niaD–gtr1*, *pyrG*–P*niaD–grx5*, or *pyrG*–P*niaD–prxA*, each flanked by 5′ and 3′ homology arms, are integrated through homologous recombination. PCR verification is shown on the right for each strain: the 5′ (lane 1) and 3′ (lane 2) junctions of the integration were examined using primer pairs pyroA-check.F/pyroA-check.R (*trxR*), or pyrG-check.F/pyrG-check.R (*gtr1*, *grx5*, *prxA*), together with gene-specific primer pairs trxR-up.F/trxR.R, gtr1-up.F/gtr1.R, grx5-up.F/grx5.R, or prxA-up.F/prxA.R. Primer sets spanning the full locus (trxR-up.F/trxR.R, gtr1-up.F/gtr1.R, grx5-up.F/grx5.R, prxA-up.F/prxA.R) distinguish recombinant transformants (lane 3) from the parental strain (lane 4); lane M indicates the DNA size marker.
